# CD73 controls Myosin II–driven invasion, metastasis, and immunosuppression in amoeboid pancreatic cancer cells

**DOI:** 10.1126/sciadv.adi0244

**Published:** 2023-10-18

**Authors:** Remi Samain, Oscar Maiques, Joanne Monger, Hoyin Lam, Juliana Candido, Samantha George, Nicola Ferrari, Leonie KohIhammer, Sophia Lunetto, Adrian Varela, Jose L. Orgaz, Felip Vilardell, Jorge Juan Olsina, Xavier Matias-Guiu, Debashis Sarker, Adrian Biddle, Frances R. Balkwill, Jim Eyles, Robert W. Wilkinson, Hemant M. Kocher, Fernando Calvo, Claire M. Wells, Victoria Sanz-Moreno

**Affiliations:** ^1^ Centre for Tumour Microenvironment, Barts Cancer Institute, Queen Mary University of London, Charterhouse Square, London EC1M 6BQ, UK.; ^2^ School of Cancer and Pharmaceutical Sciences, Kings College London, London SE1 1UL, UK.; ^3^ GSK, R&D Portfolio, Strategy and Business Insights, GSK House, 980 Great West Road, Brentford, TW8 9GS, UK.; ^4^ Oncology R&D, AstraZeneca, Cambridge CB21 6GH, UK.; ^5^ Tumour Microenvironment Team, The Institute of Cancer Research, 237 Fulham Road, London SW3 6JB, UK.; ^6^ Translational Science and Experimental Medicine, Early Respiratory and Immunology, BioPharmaceuticals R&D, AstraZeneca, Cambridge, UK.; ^7^ Centre for Cell Biology and Cutaneous Research, Blizard Institute, Queen Mary University of London, London E1 2AT, UK.; ^8^ Instituto de Investigaciones Biomédicas Sols-Morreale CSIC-UAM, 28029 Madrid, Spain.; ^9^ Department of Pathology, University Hospital Arnau de Vilanova, University of Lleida, Lleida, Spain.; ^10^ Department of Surgery, University Hospital Arnau de Vilanova, University of Lleida, Lleida, Spain.; ^11^ IRBLLEIDA, IDIBELL, University Hospita of Bellvitge, CIBERONC, Lleida, Spain.; ^12^ Centre for Tumour Biology, Barts Cancer Institute, Queen Mary University of London, Charterhouse Square, London EC1M 6BQ, UK.; ^13^ Barts and the London HPB Centre, The Royal London Hospital, Barts Health NHS Trust, London, UK.; ^14^ Instituto de Biomedicina y Biotecnologia de Cantabria, c/ Albert Einstein 22, E39011 Santander, Spain.

## Abstract

Pancreatic ductal adenocarcinoma (PDAC) has a very poor prognosis because of its high propensity to metastasize and its immunosuppressive microenvironment. Using a panel of pancreatic cancer cell lines, three-dimensional (3D) invasion systems, microarray gene signatures, microfluidic devices, mouse models, and intravital imaging, we demonstrate that ROCK–Myosin II activity in PDAC cells supports a transcriptional program conferring amoeboid invasive and immunosuppressive traits and in vivo metastatic abilities. Moreover, we find that immune checkpoint CD73 is highly expressed in amoeboid PDAC cells and drives their invasive, metastatic, and immunomodulatory traits. Mechanistically, CD73 activates RhoA–ROCK–Myosin II downstream of PI3K. Tissue microarrays of human PDAC biopsies combined with bioinformatic analysis reveal that rounded-amoeboid invasive cells with high CD73–ROCK–Myosin II activity and their immunosuppressive microenvironment confer poor prognosis to patients. We propose targeting amoeboid PDAC cells as a therapeutic strategy.

## Teaser

CD73 controls Myosin II driven amoeboid invasion and immunosuppression in pancreatic cancer.

## INTRODUCTION

Pancreatic ductal adenocarcinoma (PDAC) remains one of the deadliest cancers, and it is estimated that it will become the second leading cause of cancer-related deaths by 2030 (*1*). The five-year survival rate remains just around 7% and more than 50% of diagnosed patients present with metastatic disease (*2*). Metastasis is the leading cause of cancer-related deaths, indicating an urgent need for advances in understanding metastasis biology (*3*).

To reach distant organs, cancer cells disseminate using different migration modes, including collective, mesenchymal-elongated, and rounded-amoeboid strategies (*4*). Modes of cell migration and tumor malignant progression are heavily influenced by the tumor microenvironment (*5*, *6*). While collective cell migration plays an important role in tissue remodeling, individual cell migration allows transport to distant sites (*7*). During cancer progression, epithelial-to-mesenchymal transition (EMT) increases the ability of cancer cells to migrate and invade (*8*). Hallmarks of EMT include down-regulation of E-cadherin and activation of mesenchymal markers such as vimentin and N-cadherin, under the control of key transcription factors such as Zeb1, Twist, and Snail (*8*).

Mesenchymal cells can show further plasticity and acquire amoeboid properties when growing in three-dimensional (3D) matrices and under confinement (*9*). While RhoC supports pancreatic cancer migration (*10*), only mesenchymal-elongated migration has been described in PDAC (*11*, *12*) and mainly in 2D systems. Pancreatic tumors display a very dense extracellular matrix (ECM) rich in collagen that favors the malignancy of cancer cells (*13*). Amoeboid behavior is mainly observed in 3D matrices or under confinement; it is characterized by a rounded morphology and high RhoA/RhoC-ROCK–dependent Myosin II activity, key to sustaining bleb-based migration (*9*, *14*). Rho–ROCK–Myosin II controls all stages of the metastatic cascade in several cancer types (*15*, *16*) including pancreatic cancer (*17*–*20*). Amoeboid behavior is also associated with stem cell features in melanoma and breast cancer (*21*, *22*). Furthermore, ROCK inhibitors (ROCKis) sensitized pancreatic cancer stem cells to gemcitabine (*23*), suggesting a role of ROCK-driven signaling in both cell stemness and chemoresistance, two markers of PDAC aggressiveness. To disseminate and successfully metastasize, cancer cells need not only to become migratory but also to evade immunity. We have shown that amoeboid melanoma cells reprogram the immune microenvironment via secretion of immunosuppressive factors (*24*–*26*). Pancreatic tumors are characterized by a highly immunosuppressive microenvironment composed of cancer-associated fibroblasts (CAFs), tumor-associated macrophages (TAMs), myeloid-derived suppressive cells (MDSCs), and T-regulatory lymphocytes (*27*, *28*). PDAC tumors are also characterized by very low CD8 T lymphocyte recruitment and activation (*27*, *28*). Nevertheless, whether amoeboid cells exist in pancreatic cancer and how/if they exert immunosuppressive checkpoint control is currently unclear. In the present study, we sought to understand whether amoeboid behavior is a feature of pancreatic cancers and how they interact with their tumor microenvironment to support their aggressive behavior.

## RESULTS

### Individual pancreatic cancer cells display amoeboid features, express EMT genes, and are highly invasive

An amoeboid cellular state requires a transcriptional program compatible with the EMT spectra (*8*, *21*). Using gene set enrichment analysis (GSEA) analysis of amoeboid melanoma cell genes (*29*), we observed that amoeboid melanoma cells with high ROCK-Myosin activity were strongly enriched in genes of the EMT signature (fig. S1A). We hypothesized that in epithelial tumors, the classical mesenchymal behavior described in 2D could possibly be an amoeboid state in 3D. Therefore amoeboid could be at the opposite end of the spectra when compared to an epithelial state.

**Fig. 1.**
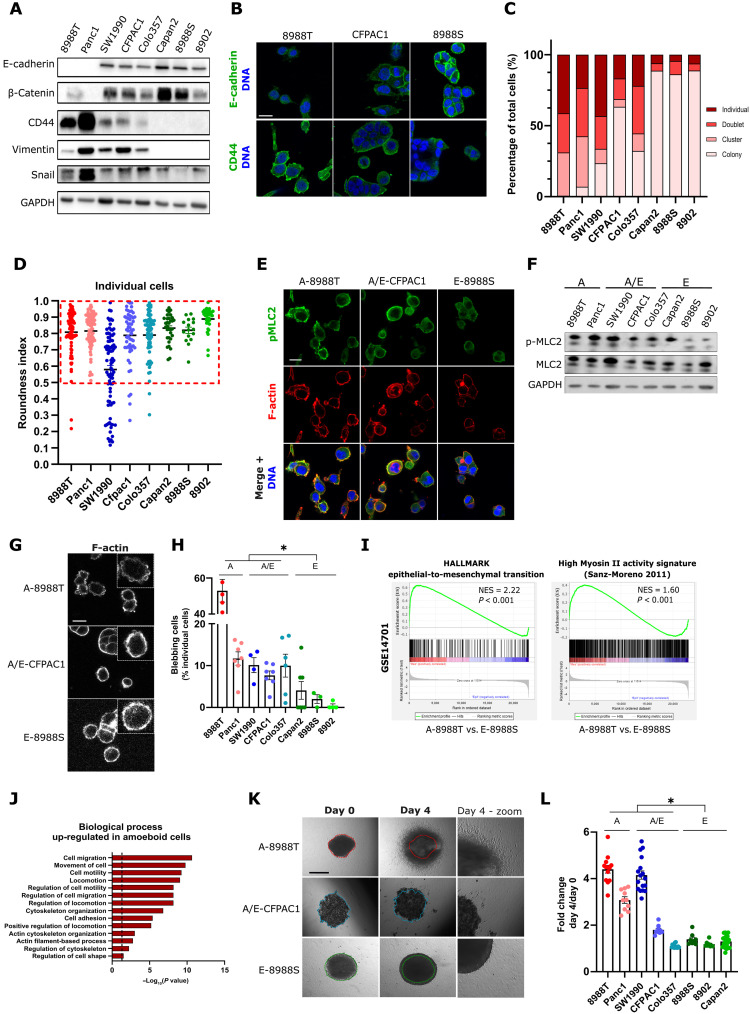
Individual pancreatic cancer cells display amoeboid features, express EMT genes, and are highly invasive. (**A**) Representative immunoblots of E-cadherin, β-catenin, CD44, vimentin, Snail, and glyceraldehyde-3-phosphate dehydrogenase (GAPDH). (**B**) E-cadherin and CD44 confocal images in PaTu8988T, CPFAC1, and PaTu8988S cells (scale bar, 20 μm). (**C**) Quantification of individual cellular event repartition (*n* ≥ 208 cells) of PaTu8988T, Panc1, SW1990, CFPAC1, Colo357, Capan2, PaTu8988S, and PaTu8902 cell lines. (**D**) Quantification of individual cellular morphology of individual cells (*n* ≥ 15 cells) of PaTu8988T, Panc1, SW1990, CFPAC1, Colo357, Capan2, PaTu8988S, and PaTu8902 cell lines. (**E**) pMLC2 and F-actin confocal images in PaTu8988T, CFPAC1, and PaTu8988S cells (scale bar, 20 μm). (**F**) Representative immunoblots of pMLC2, total MLC2, and GAPDH. (**G**) F-actin confocal images in PaTu8988T, CFPAC1, and PaTu8988S cells (scale bar, 20 μm). (**H**) Quantification of the proportion of blebbing cells (*n* ≥ 4). (**I**) GSEA plots showing enrichment of “EMT” (I) and “High–Myosin II activity” (J) gene signatures in PaTu8988T cells compared to PaTu8988S cells. (**J**) Enrichment analysis showing Gene Ontology biological process up-regulated in amoeboid cells compared to epithelial cells. (**K**) Representative images of PaTu8988T, CFPAC1, and PaTu8988S spheroids at day 0, day 4, and day 4 with high magnification (scale bar, 500 μm). (**L**) Quantification of PDAC spheroid growth invasion (n ≥ 3, each dot represents a spheroid). [(D), (H), and (L)] graphs show mean ± SEM. *P* value to compare the proportion of blebbing cells (H) was calculated using Mann-Whitney test. *P* value to compare spheroid growth invasion (L) was calculated using Student’s test.

To explore amoeboid traits in pancreatic cancer cells, a panel of eight human PDAC cell lines from different origins (primary or metastatic sites) and with different mutation profiles (Table 1) was used. We assessed the expression of epithelial or amoeboid/mesenchymal markers (assuming mesenchymal genes are also a characteristic of amoeboid cancer cells) and how that related to their cytoskeletal organization and their cell-cell interactions. Cell lines displayed different levels of EMT proteins, with two lines expressing amoeboid/mesenchymal markers only (CD44, vimentin, and Snail), three lines expressing epithelial markers only (E-cadherin and β-catenin), and both markers for three of them (Fig. 1A). We observed that PaTu8988T expressed higher levels of CD44, Nanog, KLF4, Oct4, and CD13, markers linked to cancer stemness properties (*30*, *31*), compared to PaTu8988S (fig. S1B). These cells were isolated from the same patient and have a similar genetic background (*32*). Next, cells were grown on collagen I matrices to recapitulate the complex matrices found in tumors. Protein levels of key markers were confirmed by immunofluorescence (Fig. 1B). Cells were then classified as “individual,” “doublets,” “cluster,” or “colony” (fig. S1C; see Materials and Methods). We observed heterogeneous behaviors, with important differences in the proportion of individual cells. Epithelial cells organized mostly as colonies with a minimal proportion of individual cells, amoeboid/mesenchymal cells that mostly grow as individual-doublet cells, and a mixed population of cells growing both as colonies and as individual cell behaviors for cells expressing both markers (Fig. 1C). Unexpectedly, individual cells on collagen changed their morphology and were rounded rather than elongated (compared to what we observed on plastic for individual cells; fig. S1D). Globally, cells growing as individual cells were rounded, a feature of amoeboid cells (roundness > 0.5, dashed box; Fig. 1D). Individual cells also displayed high Myosin II activity as measured by phosphorylation of myosin light chain 2 (pMLC2) immunofluorescence (Fig. 1E), confirming that individual cells expressing EMT markers were amoeboid. We therefore classified the cell lines as amoeboid (A), individual cells expressing EMT markers; epithelial (E), cells in colonies expressing mainly epithelial markers; and amoeboid/epithelial (A/E) showing mixed behavior (Fig. 1E). We confirmed that a higher proportion of individual cells (and low proportion of cells forming colonies) in a given population correlated with higher levels of pMLC2 by Western blot (Fig. 1F and fig. S1E). Blebs in amoeboid cells are a hallmark of high Myosin II activity, but they are also functionally crucial for cell migration (*5*), while they act as prosurvival oncogenic signaling platforms (*33*). The same cell lines, showing individual cell behavior and cell rounding, displayed membrane blebbing (Fig. 1, G and H). Globally, these results show that even within PDAC heterogeneous cell lines, amoeboid behavior is prevalent in individual cells. Individual mesenchymal PDAC cancer cells with spindle morphologies have been described mainly in 2D, but when grown in pliable collagen I matrices, they adopt a rounded morphology with expression of mesenchymal markers, high Myosin II activity, and characteristic membrane blebbing, indicating that they are amoeboid cancer cells. Epithelial-to-amoeboid transition is therefore possible in PDAC.

**Table 1.** Origin and mutation profiles of PDAC cell lines. WT, wild type; HD, homozygous deletion; N.A., not available/determined.

Cell line	Origin	*KRAS*	*TP53*	*CDKN2A*	*SMAD4*
PaTu8988T	Liver metastasis	12 V	282 W	N.A.	N.A.
Panc1	Primary tumor	12D	273H	HD	WT
SW1990	Spleen metastasis	12D	WT	N.A.	N.A.
CFPAC1	Liver metastasis	12 V	242R	WT	HD
Colo357	Lymph node metastasis	WT	WT	Meth	HD
Capan2	Primary tumor	12 V	WT	WT	WT
PaTu8988S	Liver metastasis	12 V	282 W	N.A.	N.A.
PaTu8902	Primary tumor	12 V	176S	WT	WT

Amoeboid melanoma cells are preferentially localized at the periphery of human and mouse tumors (*25*, *26*, *34*) and have a distinctive transcriptome (*29*). To explore whether amoeboid PDAC cells are in a transcriptional state that resembles that of amoeboid melanoma cells, GSEA was performed in a published transcriptional signature for PDAC PaTu8988T (that we defined as amoeboid cell line) and PaTu8988S (epithelial E cell line) cells. Amoeboid pancreatic cancer cells were enriched in genes related to EMT and related to amoeboid melanoma cells (Fig. 1I). In addition, PDAC metastasis genes are enriched in amoeboid PDAC cells, with particular enrichment of gene signatures that characterize cells at the periphery of pancreatic tumors (fig. S1F). Gene ontology analysis of 637 genes commonly up-regulated in two amoeboid pancreatic cancer cell lines compared to two epithelial cell lines (fig. S1G) highlighted cell migration and cytoskeleton organization as the key biological processes up-regulated in amoeboid PDAC (Fig. 1J). These data suggest that invasive and metastatic behaviors in PDAC could be related to amoeboid transcriptional and cytoskeletal features.

As tumors become aggressive, they grow and invade. To assess PDAC cell invasive growth abilities, we cultured cells as spheroids embedded in collagen I complex matrices and observed invasive growth of all amoeboid cell lines and two of three amoeboid/epithelial mixed cell lines. In contrast, all epithelial cells were unable to invade even after 4 days in this experimental setting (Fig. 1, K and L). To measure purely individual cell invasive potential, the same cells were challenged to 3D invasion assays (fig. S1H), and after 24 hours, we observed similar invasive patterns (fig. S1I). Overall, our data show that an enrichment in amoeboid behavior in pancreatic cancer cells is linked to their invasiveness.

### ROCK-dependent Myosin II activity controls PDAC migration and 3D invasion

**Fig. 2.**
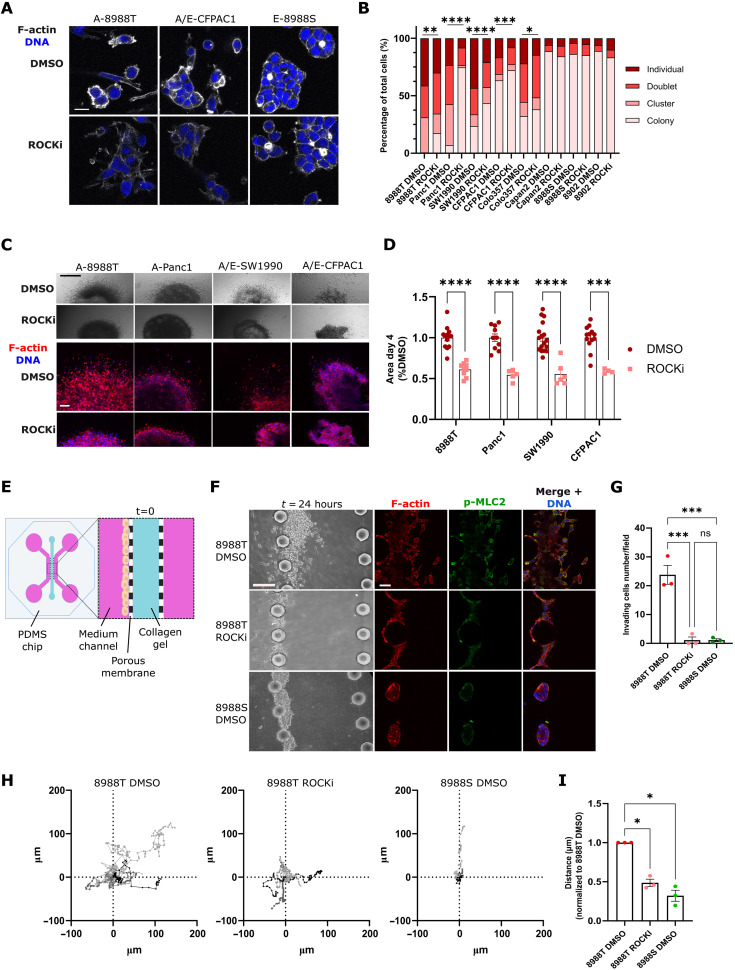
PDAC migration and invasion rely on ROCK. (**A**) F-actin confocal images in PaTu8988T, CFPAC1, and PaTu8988S cells treated with 1 μM GSK269962A or DMSO (control) for 24 hours (h) (scale bar, 20 μm). (**B**) Quantification of cellular event repartition of PDAC cell lines treated with 1 μM GSK269962A (ROCKi) or dimethyl sulfoxide (DMSO) for 24 hours (*n* ≥ 129 cells). (**C**) Representative brightfield (top) (scale bar, 500 μm) and F-actin–stained (bottom) (scale bar, 100 μm) images of spheroids treated with 1 μM GSK269962A (ROCKi) or DMSO at day 4. (**D**) Quantification of growth invasion of A and A/E spheroids treated with 1 μM GSK269962A or DMSO (*n* ≥ 3, normalized to DMSO-treated spheroid area at day 4; each dot represents a spheroid). (**E**) Schematic of PDMS chip design (see Materials and Methods). (**F**) Representative brightfield, F-actin, pMLC2, and DNA stained pictures of PaTu8988T treated with DMSO, 1 μM GSK269962A (ROCKi), or PaTu8988S treated with DMSO, invading in the collagen channel of the PDMS chip (scale bar, 100 μm). (**G**) Quantification of the average number of invading cells per field for PaTu8988T treated with DMSO, ROCKi, or PaTu8988S treated with DMSO (*n* = 3, each dot represents an independent chip). (**H**) Manual tracking of PaTu8988T cells treated with DMSO or 1 μM GSK269962A and PaTu8988S cells treated with DMSO moving on collagen I for 16 hours. (**I**) Quantification of the average distance of individual migrating cells (*n* = 3, each dot represents an independent experiment). [(D), (G), and (I)] graphs show mean ± SEM. *P* values to compare the proportion of individual cells (B) were calculated using Fisher’s exact test. *P* values to compare spheroid growth invasion (D) and numbers of invasive cells (G) were calculated using one-way ANOVA with Tukey’s multiple comparisons test. *P* values to compare cell distance (I) were calculated using one-way ANOVA with Holm-Šídák’s multiple comparisons test.

Myosin II activation can be regulated by multiple kinases including ROCK, MLCK, and MRCK (*35*). In melanoma, we have previously shown that amoeboid invasion strongly relies on ROCK activity (*25*, *26*, *34*). To investigate the role of ROCK–Myosin II activity in PDAC invasive behavior, cells growing on collagen matrices were treated with ROCKi GSK269962A, a highly potent and selective inhibitor of ROCK1/2 (*36*). MLC2 phosphorylation was decreased by ROCK inhibition, confirming that this kinase plays a key role in regulating Myosin II activity in individual PDAC cells (fig. S2, A, and B). We observed that in amoeboid and amoeboid/epithelial cells, ROCK inhibition resulted in a reduction in the proportion of individual cells and an increase in cells in groups (Fig. 2, A and B). These data suggest that ROCK inhibition in PDAC cells causes a partial reversion to an epithelial state. After ROCKi, we detected lower Myosin II activity in epithelial cells, but no further change was measured regarding their individual/colony status (Fig. 2, A and B). These results suggest that ROCK–Myosin II supports individual migratory features in PDAC cells particularly amoeboid behavior.

Next, PDAC spheroids embedded into collagen I were treated with ROCKi and allowed to grow and invade. After 4 days of ROCK inhibition, we quantified a drastic decrease in invasive growth in all cell lines tested (Fig. 2, C, and D). Using 3D collagen assays, we confirmed that ROCK inhibition decreased the individual invasion index of all amoeboid and amoeboid/epithelial PDAC cell lines tested (fig. S2, C, and D). Alternatively, we used a microfluidic system (*37*) to quantify invasion into collagen I of PaTu8988T and PaTu8988S cells, as well as PaTu8988T treated with ROCKi (Fig. 2E). Using this model, we confirmed that PaTu8988T amoeboid cells have strong invasion abilities compared to PaTu8988S epithelial cells and that these abilities were blocked in the presence of ROCKi (Fig. 2, F and G). To assess the role of ROCK purely on random cell migration, we tracked cells moving on top of collagen matrices using time-lapse video microscopy. Cell tracking revealed that amoeboid cells were highly motile compared to epithelial cells, while ROCK inhibition ablated random cell migration (Fig. 2, H and I, and movies S1 and S2). In summary, this set of data demonstrates the key role of ROCK–Myosin II activity during PDAC cell migration in collagen I, 3D invasion, and 3D invasive growth.

### ROCK–Myosin II controls invasion and metastasis in vivo in PDAC

**Fig. 3.**
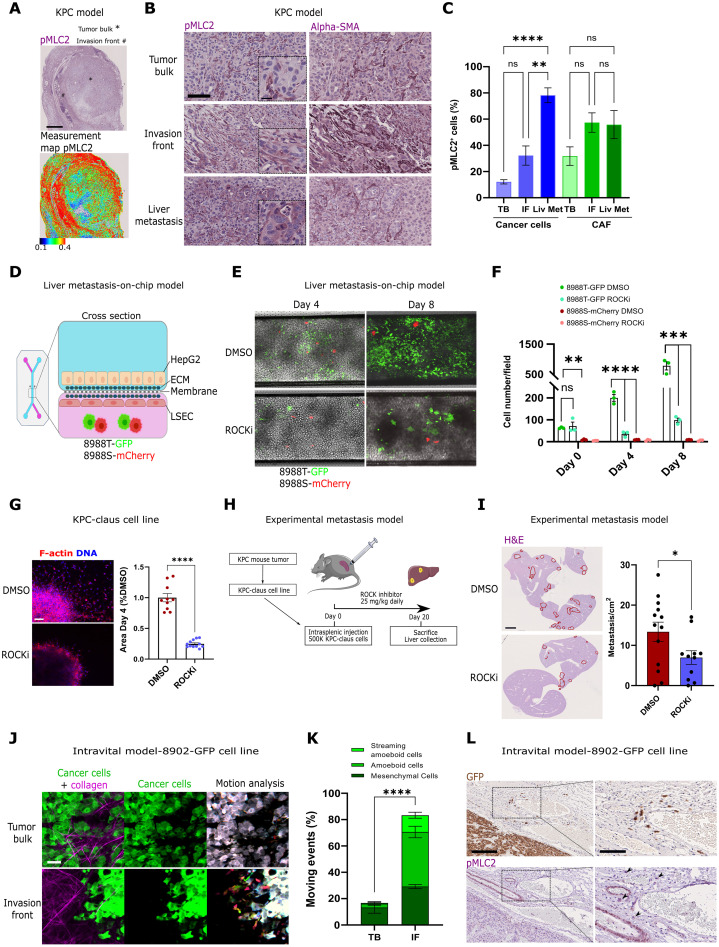
ROC–Myosin II controls invasion and metastasis in vivo. (**A**) Representative pMLC2 immunostainings (top) and intensity map (bottom) showing tumor bulk or tumor body (TB) and invasion front (IF) areas of KPC primary tumors (scale bar, 1 mm). (**B** and **C**) Representative pMLC2 and α–smooth muscle actin (α-SMA) immunostainings (B) [scale bars, 100 μm and 25 μm (inset)] and quantification of the proportion of pMLC2-positive cancer cells and cancer-associated fibroblasts (CAFs) (C) (*n* ≥ 4) within KPC TB, IF, and spontaneous liver metastasis. (**D**) Schematic of the liver metastasis-on-chip model (see Materials and Methods). (**E**) Representative pictures of attached and growing cells on the metastasis-on-chip after 4 or 8 days, upon DMSO or 1 μM GSK269962A (ROCKi). (**F**) Quantification of the average number of PaTu8988T-GFP and PaTu8988S-mCherry cells per field at days 0, 4, and 8 (*n* = 3). (**G**) Representative F-actin images (scale bar, 100 μm) and quantification of growth invasion of KPC-claus spheroids treated with 1 μM GSK269962A (ROCKi) or DMSO (*n* = 3, normalized to DMSO-treated spheroids). (**H**) Schematic of the experimental metastasis experiment protocol. (**I**) Representative H&E staining of mouse livers (left) (scale bar, 2 mm) and liver metastasis number quantification (right) (*n* = 11 to 13 mice per group) from C57Bl/6 mice intrasplenically injected with KPC-claus cells and treated with GSK269962A (25 mg/kg) or vehicle daily. (**J** and **K**) Representative images (J) and quantification of moving cells (K) in intravital PaTu8902-GFP tumors (scale bar, 50 μm; *n* = 3 tumors). (**L**) Representative GFP and pMLC2 immunostainings of fixed tissue from intravital PaTu8902-GFP model tumor invasion front (left: scale bar, 250 μm; right: scale bar, 125 μm). [(C), (F), (G), (I), and (K)] graphs show mean ± SEM. *P* values to compare the percentage of pMLC2-positive cells (C) were calculated using one-way ANOVA with Tukey’s multiple comparison test. *P* values to compare chip cell numbers (F) and moving events (K) were calculated using two-way ANOVA with Sidak’s multiple comparison test. *P* values to compare spheroid invasive growth (G) and metastasis number (I) were calculated using Student’s *t* test with Welch’s correction.

KPC mice (*Pdx-1-Cre; LSL-Kras^G12D/+^; LSL-Trp53^R172H/+^*) are physiologically close to human PDAC, spontaneously develop metastasis to the liver (*38*), and were next used to study Myosin II–dependent invasive behavior in vivo. Using pMLC2 immunostaining in the pancreatic tumors from these mice, we observed a global increase in Myosin II–high activity in tumors compared to normal pancreas, even at early stages (fig. S3A). We also observed an increase in Myosin II activity in pancreatic cancer cells located at the invasive front (IF) of tumors (Fig. 3, A and B). Supervised machine learning was used to automatically identify cancer cells and CAFs (fig. S3B). The proportion of cancer cells with high Myosin II activity increased from 12 to 32% between tumor bulk or tumor body (TB) and IF. Moreover, the proportion of Myosin II–high cells reached 78% in spontaneous liver metastasis (Fig. 3, B and C). CAFs are also very contractile cells in the tumor microenvironment (*29*, *39*, *40*). Fibroblasts with high Myosin II activity were found in both TB and IF, with an increase from 32 to 57% for these compartments, respectively (Fig. 3, B and C). However, the proportion of fibroblasts with high Myosin II activity was not further increased in liver metastasis, even if the proportion of cancer cells and fibroblasts were similar in all compartments (fig. S3C). Within the primary tumor, we observed that the proportion of E-cadherin^+^ cells was reduced at the IF (fig. S3D). These data suggest that cancer cells harboring Myosin II^high^ activity and E-cadherin^low^ levels are present in the IF of PDAC tumors and later in metastatic lesions.

To evaluate the role of ROCK–Myosin II pathway in liver metastasis formation, we developed a microfluidic model of liver metastasis-on-chip to mimic how circulating pancreatic cancer cells would attach to liver sinusoidal endothelial cells (LSECs) and survive in that liver-like microenvironment. A total of 250 labeled cancer cells were loaded into a medium flow and allowed to attach to endothelial cells in the bottom channel, while HepG2 cells, hepatocyte-like cells, were plated on the top channel (Fig. 3D). At day 0, we observed that PaTu8988T-GFP (green fluorescent protein) cells were more capable of attaching to LSEC compared to PaTu8988S-mCherry cells, independently of ROCK inhibition (Fig. 3F). After 4 and 8 days, we confirmed that amoeboid cells can preferentially proliferate within endothelial cells exposed to hepatocyte cell-secreted factors (Fig. 3, E and F). Moreover, the outgrowth of PaTu8988T cells in this environment was largely blocked by ROCKi (Fig. 3, E and F). To confirm the role of ROCK signaling in vivo, we used the KPC-claus cell line, derived from a KPC tumor. We characterized this cell line as A/E, with expression of both epithelial and EMT markers (fig. S3E), and strong invasion abilities in both spheroid invasive growth and 3D invasion assay (Fig. 3G and fig. S3, F and G). These invasive abilities were compromised after ROCKi treatment (Fig. 3G and fig. S3, F, and G). We next performed intrasplenic injections of KPC-claus into C57Bl/6 immunocompetent mice (Fig. 3H), allowing rapid dissemination of tumor cells to the liver through the portal circulation (*41*). Mice treated with ROCKi presented a substantial reduction in the number of liver metastasis when compared to control mice (Fig. 3I). We next purified liver metastasis and generated a cell line from such lesions (KPC-claus-LivMet; fig. S3H). Immunofluorescence analysis revealed that this liver metastatic cell line was characterized by an increase of pMLC2 and CD44 abundance and a decrease of E-cadherin levels compared to the parental cell line (fig. S3, H and I). We confirmed these results by Western blot, showing an increase of pMLC2 and a decrease of epithelial markers (fig. S3J), demonstrating a transition towards a more amoeboid state. These data confirm a functional role of ROCK–Myosin II in metastasis formation of pancreatic cancer in vivo and an enrichment/selection of amoeboid populations during liver metastasis.

Cancer cell lines in laboratory culture are normally established from the bulk of a tumor. We have previously described how the invasive fronts (IFs) of melanomas are enriched in amoeboid migratory cells, independently of their phenotype in the TB (*24*, *26*). We next investigated whether PDAC tumor cells would adopt amoeboid strategies in vivo, once they reach the edge of the tumor, even if their behavior was mainly epithelial on collagen cultures (and in the TB). For that purpose, we injected subcutaneously GFP-labeled PaTu8902 cells, with a very small proportion of individual cells on collagen I matrices in vitro (Fig. 1E), into CD-1 nude mice and analyzed cancer cell movement within the tumor using intravital microscopy. While, in the TB, there was very little migration (the main in vitro behavior is colony-forming), we observed that most of the cellular movement took place at the IF in the time scales analyzed. We measured a strong enrichment in amoeboid migratory behaviors (both individual and streaming amoeboid) at the edge of the tumor compared to TB (Fig. 3, J and K, and movies S3 and S4). Once tumors were fixed, we measured high pMLC2 activity at the IF of these tumors (fig. S3K), as well as the presence of rounded, individual GFP-positive and pMLC2-high cancer cells invading the surrounding healthy tissue (Fig. 3L). These data indicate that PDAC cells are plastic in vivo and that, at the edge of tumors within the matrix, amoeboid migration is prominent.

### CD73 in amoeboid pancreatic cancer cells controls an immunomodulatory secretome

**Fig. 4.**
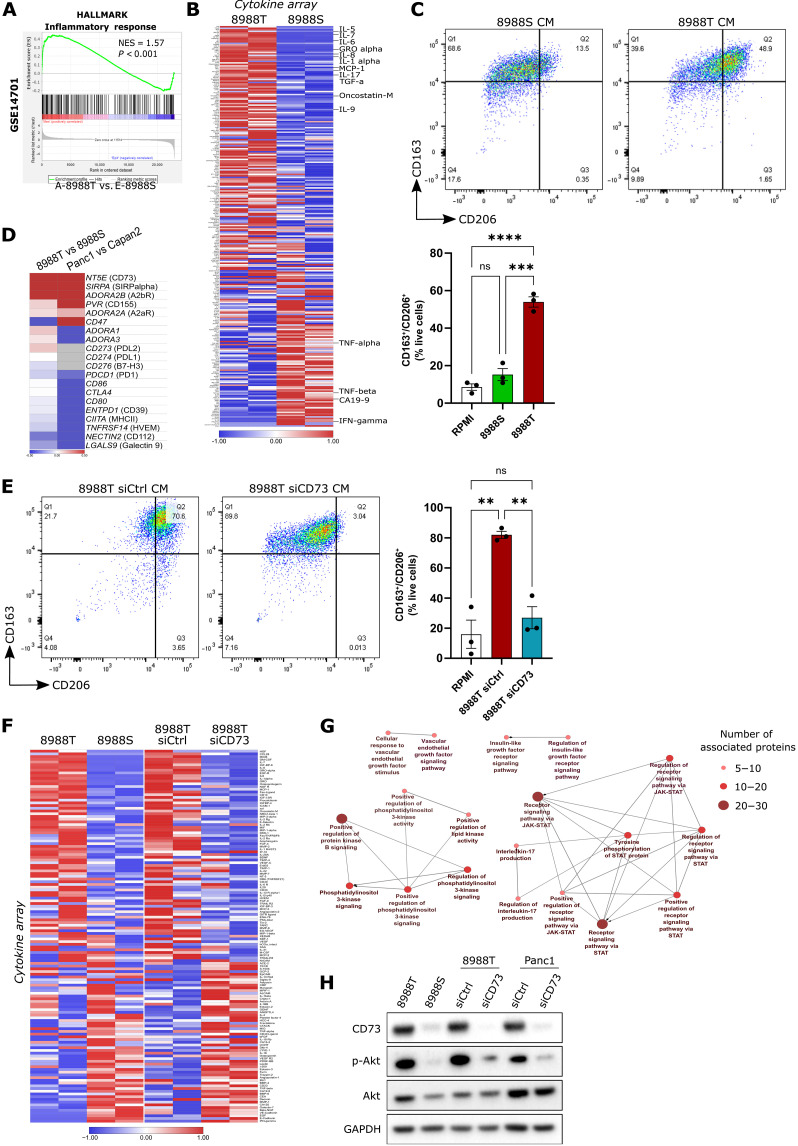
CD73 in amoeboid pancreatic cancer cells controls an immunomodulatory secretome. (**A**) GSEA plots showing enrichment of “Inflammatory response” gene signature in PaTu8988T cells compared to PaTu8988S cells. (**B**) Heatmap of secreted factors differentially enriched in conditioned media (CM) from PaTu8988T and CM from PaTu8988S. Blue and red indicate the lowest and highest expression levels, respectively (*z*-score scale). (**C**) Representative dot plots from one donor (top) and quantification of %CD163^+^CD206^+^ macrophages after treatment with RPMI culture media only, CM from PaTu8988S, and CM from PaTu8988T (bottom) (*n* = 3 different healthy donors). (**D**) Heatmap of differential expression of genes encoding for common immune checkpoints in PaTu8988T versus PaTu8988S and Panc1 versus Capan2 (*z*-score scale). (**E**) Representative dot plots from one donor (left) and quantification of %CD163^+^CD206^+^ macrophages after treatment with RPMI culture media only, CM from PaTu8988T siCtrl, and CM from PaTu8988T siCD73 (right) (*n* = 3 different healthy donors). (**F**) Heatmap of secreted factors commonly regulated in CM from PaTu8988T and CM from PaTu8988S and in CM from PaTu8988T siCtrl and CM from PaTu8988T siCD73 (*z*-score scale). (**G**) Network of signaling pathways enriched with secreted factors up-regulated in PaTu8988T cells. (**H**) Representative immunoblots of CD73, p-Akt, Akt, and GAPDH. [(C) and (E)] Graphs show mean ± SEM. *P* values to compare the %CD163^+^CD206^+^ macrophages [(C) and (E)] were calculated using one-way ANOVA with Tukey’s multiple comparison test.

To reach distant organs, cancer cells need not only to invade but also to survive immune system attack. ROCK–Myosin II activation in amoeboid melanoma cells contributes to the recruitment and polarization of protumorigenic immune cell populations such as macrophages (*25*, *26*). In this way, invasive amoeboid cancer cells create an “immune protective niche” for themselves, while they escape the primary tumor. Compared to pancreatic epithelial cancer cells, amoeboid PDAC cells are enriched in genes responsible for a tumor inflammatory response (Fig. 4A). We next tested the hypothesis that amoeboid pancreatic cancer cells could also communicate more avidly with normal cells, particularly with immune cells, and corrupt them while they leave the primary tumor. Tumor-normal cell communication can be mediated by secreted factors (*25*). Using a cytokine array consisting of 274 human chemokines, cytokines, growth factors, and matrix metalloproteinases, we found that 146 proteins were highly secreted by PaTu8988T cells compared to PaTu8988S cells. PaTu8988T amoeboid cells were shown to secrete high levels of interleukins [such as interleukin-1α (IL-1α), IL-5, IL-6, IL-7, IL-8, and IL-9], as well as several chemokines involved in cancer progression (Fig. 4B). PaTu8988S epithelial cells were found to secrete high levels of interferon-γ (IFN-γ) or tumor necrosis factor–α, which are key for antitumor immune responses (Fig. 4B). From the list, many of these factors had the potential to affect several immune populations, with a number of factors linked to monocyte/macrophage recruitment and/or polarization [such as GRO-α, granulocyte-macrophage colony-stimulating factor (GM-CSF), migration inhibition factor (MIF), Monocyte Chemoattractant Protein 1/3 (MCP-1/3), Macrophage inflammatory protein 1-α (MIP1-α), MIP3-α, Oncostatin M, IL-6, Tie-2, and IL-11. To test the effects of this medium on myeloid cells, human peripheral blood mononuclear cell (PBMC)–derived monocytes were treated with conditioned media (CM) from PaTu8988T (amoeboid PDAC cell line) versus PaTu8988S (epithelial PDAC cell line) cells. We observed that PaTu8988T-derived media largely increased the proportion of CD163^+^/CD206^+^ cells (markers of protumorigenic macrophages) compared to cells treated with control media, whereas PaTu8988S-derived media had no detectable effect on these markers (Fig. 4C). These data show that amoeboid PDAC cells are able to educate macrophages via paracrine secretion.

We next investigated whether amoeboid PDAC cells could have a different gene expression profile that could contribute to their immune-modulatory properties. Using published RNA sequencing (RNA-seq) data, we analyzed gene expression of key immune checkpoints and found that the *NT5E* gene, coding for the CD73 protein, together with ADORA2A (A2aR) and ADORA2B (A2bR), encoding for adenosine receptors were up-regulated in amoeboid PDAC cells compared to epithelial PDAC cells (Fig. 4D). CD73 is a cell surface receptor found on various cancer cell types and plays a role in promoting immunosuppression by generating adenosine from adenosine triphosphate (*42*). Adenosine can then bind to adenosine receptors and exert immunosuppressive functions in the tumor microenvironment (*42*). Reducing CD73 levels using small interfering RNA (siRNA) in PaTu8988T cells, resulted in impaired macrophage polarization (Fig. 4E). Analysis of the secretome of PaTu8988T cells after *NT5E* siRNA transfection showed a substantial reduction in important secreted factors. Sixty-two cytokines, including IL-1α, IL-6, IL-8, MIF, GRO-α, MCP-1/3, GM-CSF, MIP1-α, MIP3-α, or oncostatin M, that play major roles in controlling macrophage function were commonly down-regulated in epithelial PDAC cells or in amoeboid PDAC cells with reduced CD73 levels (Fig. 4F). On the other hand, 32 were commonly up-regulated in both cases, including IFN-γ (Fig. 4F). Network analysis of processes connected to PaTu8988T amoeboid cell secretome showed that cytokine activity and related Janus kinase (JAK)/signal transducer and activator of transcription (STAT) pathways were enriched while Phosphatidylinositol 3-kinase (PI3K) signaling was among the most enriched processes (Fig. 4G and fig. S4A). We confirmed that higher PI3K-AKT pathway engagement was a characteristic of amoeboid PDAC cells compared to epithelial PDAC cells (Fig. 4H and fig. S4B). In addition, CD73 knockdown resulted in strong reduction of p-AKT, showing that CD73 is acting upstream of PI3K-AKT (Fig. 4H and fig. S4B). These data suggest that CD73 in PDAC cells could support immune escape via cytokine/chemokine secretion and via signaling through PI3K.

### CD73 stimulates PI3K-driven Rho–ROCK–Myosin II signaling and 3D invasion

**Fig. 5.**
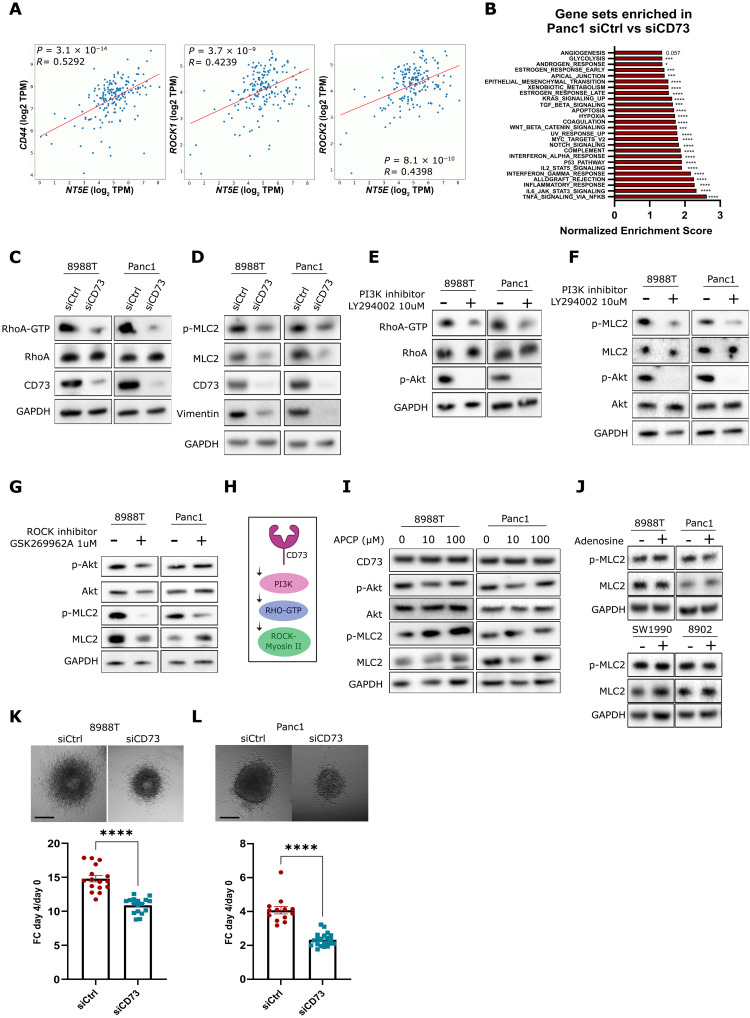
CD73 stimulates PI3K-driven Rho–ROCK–Myosin II signaling and 3D invasion. (**A**) Scatter chart showing correlation between *NT5E* and *CD44* (left), *ROCK1* (middle), and *ROCK2* (right) gene expressions in pancreatic cancer patients RNA (TCGA database). (**B**) Normalized enrichment score of hallmark gene sets identified as enriched in Panc1 siCtrl compared with siCD73 (GSE117012). (**C**) Representative immunoblots of RhoA-GTP in pulldown samples and total RhoA, CD73, and GAPDH in total lysate of PaTu8988T cells and Panc1 cells transfected with a control or *NT5E* targeting siRNA. (**D**) Representative immunoblots of pMLC2, total MLC2, CD73, Vimentin, and GAPDH in (C) cells. (**E**) Representative immunoblots of RhoA-GTP in pulldown samples and total RhoA, p-Akt, and GAPDH in total lysate of PaTu8988T cells and Panc1 cells treated with DMSO (control) or with LY294002 (10 μM for 4 hours). (**F**) Representative immunoblots of pMLC2, total MLC2, p-Akt, Akt, and GAPDH in (E) cells. (**G**) Representative immunoblots of p-Akt, Akt, pMLC2, MLC2, and GAPDH in PaTu8988T and Panc1 cells treated with 1 μM GSK269962A or DMSO for 24 hours. (**H**) Schematic of working hypothesis. (**I**) Representative immunoblots of CD73, p-Akt, Akt, pMLC2, MLC2, and GAPDH in PaTu8988T and Panc1 cells treated with APCP or DMSO for 24 hours. (**J**) Representative immunoblots of pMLC2, MLC2, and GAPDH in PaTu8988T, Panc1, SW1990, and PaTu8902 cells treated with 5 μM adenosine or DMSO (control) for 24 hours. (**K** and **L**) Representative images (top) and quantification of invasive growth (bottom) of PaTu8988T (K) and Panc1 (L) cells transfected with a control or a *NT5E* siRNA spheroids at day 4 (scale bar, 500 μm). [(K) and (L)] graphs show mean ± SEM. *P* values and *R* in (A) were calculated using Pearson correlation analysis. *P* values in (B) represent nominal *P* value calculated in GSEA software. *P* values to compare spheroid growth invasion [(K) and (L)] were calculated using Student’s *t* tests.

CD73 has previously been linked to EMT and stemness properties of ovarian, breast, liver, and gastric cancer cells (*43*–*46*). The Cancer Genome Atlas (TCGA) data analysis revealed that *NT5E* gene expression was strongly correlated with *CD44* and *ROCK1*/*ROCK2* gene expressions in pancreatic tumors (Fig. 5A), suggesting a link between CD73 and loss of epithelial features. We next used publicly available RNA-seq data comparing Panc1 amoeboid PDAC cells transfected with *NT5E* or control siRNA [GSE117012 (*47*)]. We observed that *NT5E* expression was associated with several gene sets linked to amoeboid migration and pancreatic cancer progression, such as transforming growth factor–β (TGF-β), KRAS, WNT, nuclear factor κB (NF-κB) and IL-6/JAK/STAT3 signaling, hypoxia, or EMT (Fig. 5B). This dataset, together with the secretome analysis, suggests that CD73 could be important for both invasive and immune-suppressive features of amoeboid PDAC cells. Because amoeboid melanoma cells rely on RhoA for their behavior (*14*), we confirmed that CD73 depletion in amoeboid PDAC cell lines reduced guanosine triphosphate (GTP)–bound RhoA levels (Fig. 5C and fig. S5A), accompanied by a strong reduction of MLC2 phosphorylation and a decrease in EMT marker vimentin (Fig. 5D and fig. S5B). Similar results were obtained using an alternative siRNA sequence against the CD73 gene (fig. S5, C and D). Moreover, pan-PI3K inhibition (as confirmed with decreased Akt phosphorylation levels) using LY294002 (*48*) reduced both RhoA activity (Fig. 5E and fig. S5E) and downstream MLC2 phosphorylation after 4-hour treatment (Fig. 5F and fig. S5F). On the other hand, ROCK inhibition did not affect Akt phosphorylation (Fig. 5G), suggesting that RhoA–ROCK–Myosin II are activated downstream of PI3K (Fig. 5H). Treatment with either Α,β-methylene adenosine-5′-diphosphate (APCP; to inhibit CD73 enzymatic activity) or adenosine (to stimulate adenosine signaling) did not alter p-AKT nor pMLC2 levels (Fig. 5, I and J), suggesting an adenosine-independent regulation of this pathway. PaTu8988T or Panc1 amoeboid PDAC cells grown as spheroids displayed severely impaired invasive growth after CD73 depletion (Fig. 5, K and L). These data shows that CD73 regulates invasion and PI3K-Rho-Myosin cytoskeletal remodeling, independently of its catalytic activity.

### CD73 stimulates Myosin II–dependent immunosuppression and metastatic spread in vivo

**Fig. 6.**
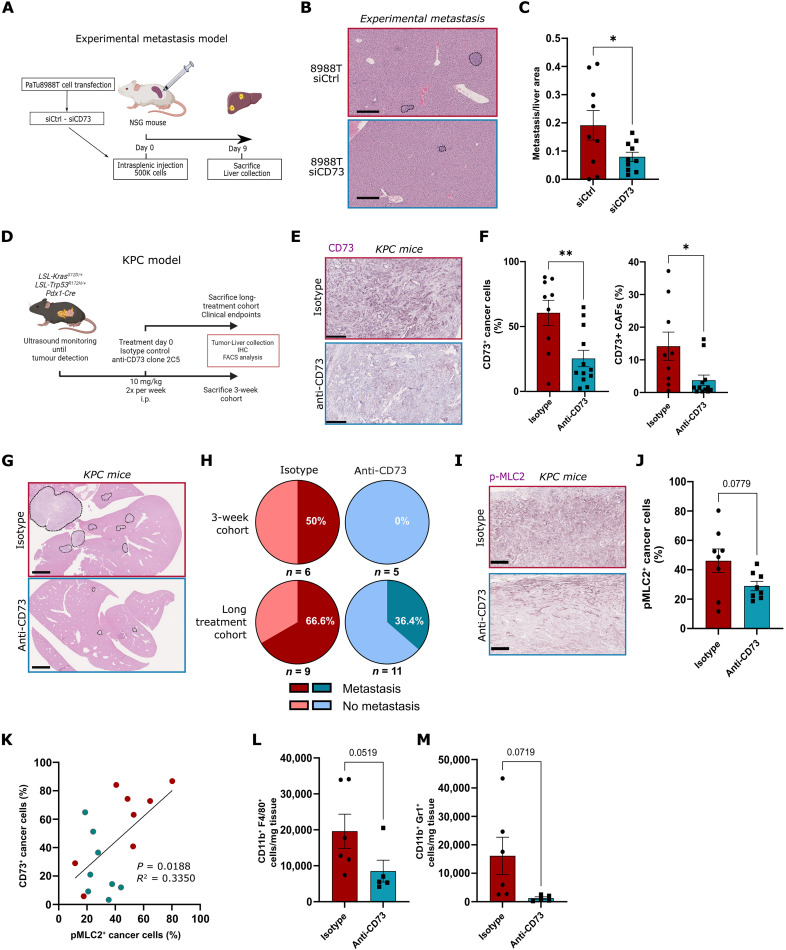
CD73 stimulates Myosin II–dependent immunosuppression and metastatic spread in vivo. (**A**) Schematic of the protocol used for experimental metastasis experiment. (**B**) Representative H&E staining of livers from NSG mice intrasplenically injected with PaTu8988T cancer cells transfected with control or *NT5E* siRNA (scale bar, 250 μm). (**C**) Liver metastasis number quantification (*n* = 9 to 10 mice per group). (**D**) Schematic of the protocol used for KPC experiment. (**E**) Representative CD73 immunostainings of KPC tumors after treatment (scale bar, 250 μm). (**F**) Quantification of the proportion of CD73-positive cancer cells and CD73-positive CAFs within KPC tumors after treatment with control isotype (*n* = 9) or anti-CD73 (*n* = 12). (**G**) Representative H&E staining of mouse livers from KPC mice after treatment (long-treatment cohort; scale bar, 2.5 mm). (**H**) Pie charts showing liver metastasis incidence in KPC mice after treatment for 3 weeks and long-treatment cohorts. (**I**) Representative pMLC2 immunostainings of KPC tumors after treatment with control isotype or anti-CD73 (scale bar, 250 μm). (**J**) Quantification of the proportion of pMLC2-positive cancer cells and CAFs within KPC tumors after treatment with control isotype (*n* = 9 mice) or anti-CD73 (*n* = 8 mice; mice with no PDAC stage tumors were excluded from the analysis). (**K**) Scatter chart showing correlation between CD73 and pMLC2 levels in cancer cells within KPC tumors. (**L** and **M**) FACS analysis of CD11b^+^F4/80^+^ macrophages (L) and CD11b^+^Gr1^+^ myeloid cells (M) per milligram of KPC tumor tissue after treatment with control isotype (*n* = 6 mice) or anti-CD73 (*n* = 5 mice). [(C), (F), (J), (L), and (M)] graphs show mean ± SEM. *P* values to compare metastatic area (C) and CD73 and pMLC2 positive cells [(F) and (J)] were calculated using Student’s *t* tests. *P* value and *R* squared in (K) were calculated using Pearson correlation analysis. *P* values to compare macrophages (L) and myeloid cells numbers (M) were calculated using Mann-Whitney test and Student’s *t* test with Welsh’s correction, respectively.

To understand the therapeutic potential of our observations using preclinical models, we evaluated the effects of blocking CD73 functions to target amoeboid invasive and immunosuppressive properties in PDAC. PaTu8988T cells injected in the mouse spleen (Fig. 6A) effectively colonized and grew in the liver, while same cells were significantly less efficient after *NT5E* siRNA transfection (Fig. 6, B and C). CD73-targeting antibodies are in active development for their use in the clinic (NCT04940286 and NCT04148937); we sought to validate our in vitro observations with preclinical validation using these antibodies.

KPC mice were monitored until tumor detection by ultrasound and treated using anti-CD73 (clone 2C5) antibody or isotype control for 3 weeks (short-treatment cohort) or until clinical end points were met (long-treatment cohort; see Materials and Methods; Fig. 6D). The 2C5 clone is able to not only bind CD73 with high affinity, inhibit its ectonucleotidase activity, and engage Fc receptors but also induce CD73 internalization (*43*). We observed that treatment with anti-CD73 antibody resulted in a strong reduction of CD73 levels in both cancer cells and CAFs (Fig. 6, E and F). Moreover, when mice were culled after only 3 weeks of treatment, none of the anti-CD73 antibody–treated mice (*n* = 5) showed liver metastasis, compared to three of six in the isotype-treated cohort (Fig. 6, G and H). In the long treatment cohort, anti-CD73 treatment reduced the incidence of liver metastasis, from 66.6 to 36.4% (Fig. 6, G and H). However, the treatment did not affect tumor growth, as we observed similar tumor volumes in both groups after 2 weeks of treatment (fig. S6A). We also verified in tumors at PDAC stage that pancreatic cancer cells had reduced Myosin II activity when tumors were treated with CD73 blocking antibody, with no effects on CAFs (Fig. 6, I and J, and fig. S6B), suggesting that CD73 has a specific role in controlling Myosin II activity in cancer cells. We observed a correlation between CD73 levels and Myosin phosphorylation in cancer cells in KPC tumors (Fig. 6K). Blocking CD73 resulted in reduced recruitment of protumorigenic immune cells. As such, fluorescence-activated cell sorting (FACS) analysis revealed decreased CD11b^+^F4/80^+^ TAMs and CD11b^+^Gr1^+^ MDSCs in the anti-CD73–treated tumors (Fig. 6, L and M, and fig. S6C), while CD4^+^ and CD8^+^ T lymphocytes were not affected (fig. S6, D and E). Analysis of publically available single-cell data from human PDAC tumors (GSE154778) (*49*) suggested that CD73 was mainly expressed in malignant cells, supporting the idea that CD73 inhibition will mainly affect cancer cells (fig. S6F). Together, these data show that blocking CD73 function reduces metastatic and immunosuppressive properties of amoeboid cancer cells in vivo.

### CD73–ROCK–Myosin II are biomarkers of human PDAC aggressiveness

**Fig. 7.**
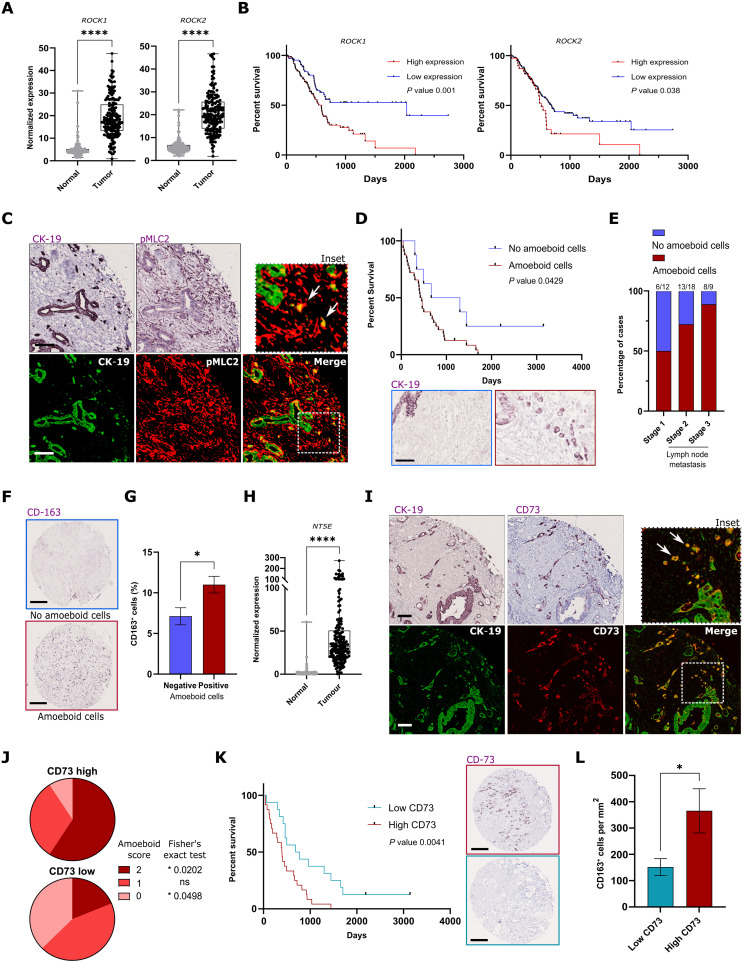
CD73–ROCK–Myosin II as biomarkers of human PDAC aggressiveness. (**A**) Normalized mRNA gene expression of *ROCK1* and *ROCK2* in normal (*n* = 200) and tumoral (*n* = 176) pancreas of patients (TCGA database). (**B**) Kaplan-Meier survival plot of 176 patients sorted according to expression of *ROCK1* and *ROCK2* (TCGA database). (**C**) Representative CK-19 and pMLC2 immunostainings (top) and pseudo-colored multiplex images (bottom) from a TMA section of human PDAC (scale bar, 100 μm). (**D**) Kaplan-Meier survival plot of 40 patients sorted according to the presence or absence of individual round CK-19–positive amoeboid cells (bottom) (scale bar, 100 μm). (**E**) Quantification of the proportion of tumors presenting amoeboid cells according to clinical stage (stage 1, *n* = 12; stage 2, *n* = 18; stage 3, *n* = 9). (**F**) Representative CD163 immunostainings of amoeboid cancer cell negative and positive tumors from TMA sections (scale bar, 250 μm). (**G**) Quantification of the percentage of CD163-positive cells in amoeboid cancer cell negative and positive sections (*n* = 49 to 100). (**H**) Normalized mRNA gene expression of *NT5E* in normal (*n* = 200) and tumoral (*n* = 176) pancreas of patients (TCGA database). (**I**) Representative CK-19 and CD73 immunostainings (top) and pseudo-colored multiplex images (bottom) from a TMA section (scale bar, 100 μm). (**J**) Pie chart showing the distribution of amoeboid score in CD73 high and low patients. (**K**) Kaplan-Meier survival plot of 48 patients sorted according to CD73 expression in cancer cells (right) (scale bar, 250 μm). (**L**) Quantification of the average number of CD163^+^ cells in CD73 high and low patients. [(A), (G), (H), and (L)] graphs show mean ± SEM. *P* values to compare gene expressions [(A) and (H)] and CD163^+^ cells [(G) and (L)] were calculated using Mann-Whitney tests. *P* values to compare survival [(B), (D), and (K)] were calculated using log-rank Mantel-Cox tests. *P* values to compare amoeboid score distributions (J) were calculated using Fisher’s exact *t* tests.

Using the TCGA public database, we observed that the up-regulation of *ROCK1/2*, *MYH9* (gene encoding myosin heavy chain)*, MYL9, MYL12A*, and *MYL12B* (genes encoding myosin light chain 2) is a common event in PDAC. *ROCK1/2* genes and Myosin II genes were highly expressed in tumor tissue compared to normal pancreas (Fig. 7A and fig. S7A). High ROCK and Myosin expression was associated with worse prognosis in PDAC patients (Fig. 7B and fig. S7B). GSEA analysis showed that human pancreatic tumors are enriched in amoeboid genes when compared to adjacent normal pancreas in two different RNA-seq databases (fig. S7C).

To further explore whether amoeboid invasive cells can be found in human PDAC biopsies, we analyzed a tissue microarray (TMA) with 48 human tumors (cohort A). We performed multiplex immunostaining using cytokeratin-19 (CK-19; as a marker of cancer cells) and pMLC2 (as a marker of amoeboid cells and Myosin II activity). We observed that pMLC2 staining was high not only in CAFs but also in individual CK19^+^ cancer cells as opposed to cancer cells forming clusters (Fig. 7C). More than half (67%) of the sections contained CK19^+^ rounded individual cells (fig. S7D) that we defined as amoeboid pancreatic cancer cells. The presence of amoeboid cancer cells was associated with worse prognosis (survival median of 437 days versus 981 days; Fig. 7D). Amoeboid cell content was not associated with a global increase of CAFs or ECM (fig. S7, E and F). Moreover, the intensity of pMLC2 staining in fibroblasts was not associated with patient survival, suggesting that contractility of cancer-associated fibroblasts, on its own, may not fully determine cancer aggressiveness (fig. S7G). In patients with lymph node metastasis (stages 2 and 3), amoeboid cells were found in 72.2 and 88.9% of cases, respectively, in comparison with only 50% cases for stage 1 patients (Fig. 7E). We next used a validation cohort (cohort B) composed of 19 human tumors. In this TMA, 9 of 19 patients (47%) presented amoeboid pancreatic cancer cells (fig. S7D). Inspecting the TCGA database (from www.proteinatlas.org/), we observed CK19^+^ rounded individual cells in 17 of 23 patients (74%; fig. S7D). These findings, obtained from three different cohorts, confirm the presence of amoeboid cancer cells and the expression of genes in the ROCK–Myosin II pathway as markers of human PDAC aggressiveness.

We next analyzed whether the tumor-suppressive immune infiltrate could also be characteristic of amoeboid invasive pancreatic tumor cells in patient biopsies. The presence of amoeboid cancer cells in cohort A sections was associated with an increase in CD163^+^ (Fig. 7, F and G) and CD68^+^ macrophages (fig. S7, H and I). We next used CIBERSORT (*50*) to evaluate the abundance of different immune cell populations using gene expression data of 145 PDAC tumors [GSE71729 (*51*)] sorted according to *ROCK1* expression levels. Tumors with high *ROCK1* were characterized by increased protumorigenic/inflammatory macrophage ratio (fig. S7J) and linked to decreased cytotoxic CD8^+^ T lymphocytes (fig. S7K). Moreover, in cohort B, patients with amoeboid cancer cells harbored increased neutrophil-to-lymphocyte ratio (fig. S7L), indicative of systemic inflammation, immunosuppression, and poor prognosis for patients (*52*).

We next sought to confirm CD73 as an amoeboid cancer cell marker in patient biopsies. *NT5E* gene was almost absent in normal pancreas but strongly up-regulated in pancreatic tumors from TCGA (Fig. 7H). Using cohort A TMA, we observed that some sections contained a large proportion of CD73^+^ individual cancer cells, while clusters of cancer cells were CD73 negative (Fig. 7I). High levels of CD73 in cancer cells were therefore associated with a higher amoeboid score in PDAC tumors (Fig. 7J). When sorting patients according to the proportion of CD73^+^ cancer cells, we found that CD73 abundance was associated with poor prognosis in patients (survival median of 396 days and 729 days for CD73 high and low, respectively; Fig. 7K). This was consistent with TCGA data showing that *NT5E* gene expression was linked to worse prognosis (fig. S7M). Moreover, we found higher number of CD163^+^ macrophages in CD73-high compared to CD73-low patient sections (Fig. 7L). CIBERSORT analysis of GSE71720 cohort confirmed that tumors with high *NT5E* expression were characterized by a strong increase of protumorigenic macrophages and decreased cytotoxic CD8^+^ T lymphocytes (fig. S7N). Collectively, the data show that CD73^+^ amoeboid cancer cells are a very aggressive population with a unique protumorigenic immune microenvironment. These invasive and immunosuppressive features confer poor prognosis to PDAC patients.

## DISCUSSION

Pancreatic cancer is a highly aggressive tumor, with most patients diagnosed with metastatic disease (*2*). Recent advances in therapeutic strategies, including immunotherapies, have failed to markedly improve overall survival for patients (*1*). Cancer cell dissemination starts early in pancreatic cancer evolution, with the emergence of clones able to leave the primary tumor (*53*). EMT has been described for PDAC, whereby cells lose cell-cell adhesions and acquire invasive properties and stem cell–like features (*11*, *12*). Individual cell migration is favored at the edge of many tumor types and seems to be important for metastasis (*7*). In 3D, cancer cells alternate between elongated-mesenchymal and rounded-amoeboid migration, depending on their physical environment (*5*, *54*), the balance between Rho guanosine triphosphatase (GTPase) signals (*10*, *14*), levels of adhesion (*4*, *55*), and the presence of inflammatory cytokines and growth factors (*14*, *24*, *26*, *56*). We show here that individual pancreatic cancer cells analyzed, when cultured in collagen matrices, are rounded, harbor a high level of Myosin II activity and blebbing activity, and are highly invasive. Such cells are in a transcriptional program akin to EMT. Therefore, we defined this population, only found in 3D settings, as amoeboid pancreatic cancer cells.

We show that PDAC cell lines that are more invasive in our in vitro assays mainly use individual migratory modes to invade, but they exhibit migratory plasticity in vivo. Using liver-on-chip, we show that amoeboid cancer cells attach/grow better to/with endothelial cells compared to epithelial cells. Moreover, their growth after attachment is strongly reduced after ROCK inhibition. In this model, ROCK inhibition did not affect amoeboid cell initial attachment to endothelial cells probably because of the low medium flow speed. However, ROCK–Myosin II signaling has been shown to mediate resistance to fluid shear stress in circulating tumor cells (*57*). Using models of spontaneous PDAC, we observed that IFs of mouse tumors, as well as metastatic areas, are enriched in cancer cells harboring high Myosin II activity. Using intravital imaging, we show that pancreatic cancer cells harboring mainly an epithelial phenotype switch to individual amoeboid migration when they reach the tumor border or IF. Moreover, elongated and amoeboid phenotypes belong to the EMT spectrum of behaviors because we show that amoeboid cells are strongly enriched in what has been coined as “mesenchymal” or EMT genes. We confirmed that amoeboid migration of these cells is ROCK dependent because the inhibition of the pathway blocks cell invasion and growth in vitro not only in many different assays but also in an experimental metastasis mouse model.

ROCK–Myosin II activity in melanoma cells has been linked to the induction of protumorigenic immune populations such as TAMs and regulatory T lymphocytes (*25*, *26*). Pancreatic tumors are nonimmunogenic and respond poorly to classic immunotherapies (*58*). In the current study, we find that the cytoskeleton of cancer cells controls two key aspects of metastatic dissemination: cell invasion and immune evasion. Particularly, we have identified the enzyme ecto-5′-nucleotidase, or CD73, as a new amoeboid marker involved in immune escape. CD73 is a membrane-bound immune checkpoint involved in the conversion of adenosine monophosphate (AMP) into immunosuppressive adenosine (*42*). We show a direct effect of amoeboid cell secretion (without purifying exosomes or extracellular vesicles) on macrophage polarization. This polarization is reduced when CD73 is depleted. CD73-positive cancer exosomes can alter T cell functions and affect immunomodulation (*59*). Nevertheless, cytokines directly secreted by amoeboid cells could be immunomodulatory, while future work will be needed to understand the role of exosomes derived from amoeboid PDAC cells. CD73 is overexpressed in various solid tumors including pancreatic cancer (*47*). Previous work has linked CD73 abundance and adenosine signaling in cancer cells to stemness, EMT (*22*, *43*–*46*), metastasis (*60*), and chemoresistance (*61*). The contribution of CD73 to tumor progression has mainly been studied through its enzymatic activity and the production of immunosuppressive adenosine from extracellular AMP (*42*). We find that this noncanonical function of CD73 supports PI3K signaling and downstream RhoA–Myosin II–dependent invasion, independently of its catalytic activity. CD73 noncatalytic roles have been previously reported (*61*). On the other hand, in hepatocellular carcinoma, CD73 activates Rap1, leading to P110β-PI3K signaling and AKT phosphorylation (*62*). Blebbing triggers the formation of plasma membrane–proximal signaling hubs that confer anoikis resistance. Specifically, in melanoma amoeboid cells, blebbing generates plasma membrane contours that recruit curvature-sensing septin proteins as scaffolds for constitutively active mutant NRAS and effectors. These signaling hubs activate ERK and PI3K well-established promoters of prosurvival pathways. (*33*). Moreover, active Akt can phosphorylate the deleted in liver cancer 1 protein (a GTPase activating protein for RhoA), leading to an inhibition of its activity and therefore increasing levels of RhoA-GTP (*63*). All these mechanisms could further support cross-talk between CD73, PI3K, and Rho-ROCK.

CD73 in amoeboid cancer cells controls a tumor-promoting secretome. Targeting CD73 via siRNA or using blocking antibodies in the KPC model resulted in a remarkable decrease in liver metastasis incidence, correlated with reduced Myosin II activity. However, CD73 targeting did not affect primary tumor, suggesting that a combination therapy would be more efficient for patients to target both tumor growth using chemotherapy and its spread using anti-CD73 antibodies. Blocking CD73 was associated with a reduction in protumorigenic immune cells, while T lymphocytes were not affected by the treatment. CD73 distinguishes regulatory T cell (T_reg_) lymphocytes from other T cells in the lymphoid compartment in mice (*64*). Therefore, blocking of CD73 in T_reg_ lymphocytes could potentially reduce immunosuppression. Nevertheless, using publically available single-cell data, we find that CD73 is mainly expressed by cancer cells in human PDAC tumors, indicating that CD73-blocking therapy could target aggressive cancer cells with less effects on other cell populations within tumor microenvironment.

When analyzing human biopsies, we confirmed that amoeboid cancer cells exist in human PDAC (CK19^+^ individual rounded cells with Myosin^high^ and CD73^+^). The presence of such cells was associated with worse prognosis, while they were found in ~90% of stage 3 patients of our cohort. Such cells were already found in early-stage tumors, suggesting that amoeboid cells may arise early in disease but are selected for during progression. Actomyosin contractility plays an important role in the tumor microenvironment, and the activation of the ROCK/Myosin II axis in cancer-associated fibroblasts has been shown to favor cell invasion in different cancer types (*29*, *39*, *40*, *65*–*68*). Although PDAC tumors contain a large number of CAFs, Myosin II activity alone in these fibroblasts was not correlated with patient survival. This suggests that a further set of fibroblast markers may be required to assess their contribution to the aggressiveness of the disease (*6*, *69*). We also describe here how the presence of amoeboid cancer cells in PDAC correlates with a specific protumorigenic immune infiltrate, composed of macrophages and neutrophils, while cytotoxic T cells are reduced. We suggest a decrease in the highly aggressive amoeboid cancer cell populations as a marker of response in the ongoing clinical trials using CD73 targeting drugs (NCT04940286 and NCT04148937) in PDAC. ROCKi (*70*) could also be considered in this context.

**Fig. 8.**
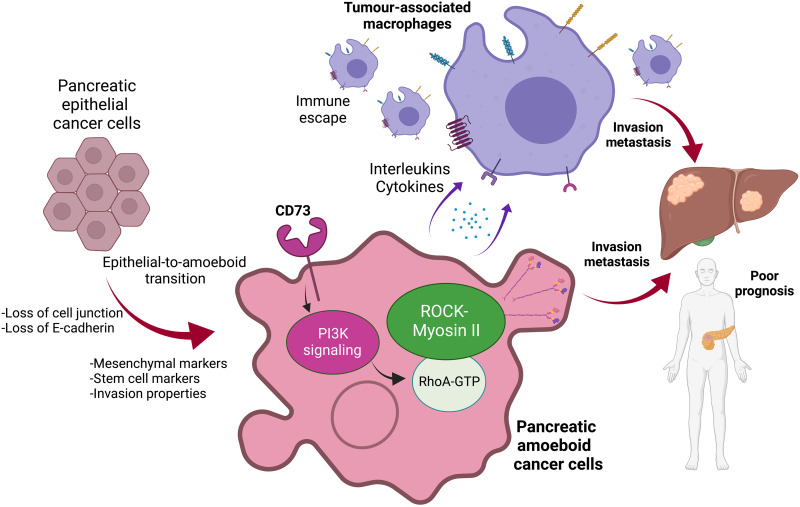
CD73 and ROCK controls Myosin II–driven amoeboid invasion and immunosuppression in pancreatic cancer. In 3D environments, as well as in the IFs of tumors, epithelial pancreatic cancer cells undergo epithelial-to-amoeboid transition, with a loss of E-cadherin and a loss of cell-cell junctions. The amoeboid state is characterized by an increase in the proportion of individual cells with high levels of mesenchymal markers, stem cells markers, and Myosin phosphorylation. ROCK-dependent phosphorylation of Myosin allows amoeboid cancer cells to invade into collagen matrices. Amoeboid pancreatic cancer cells harbor elevated levels of CD73, driving PI3K activation and RhoA/ROCK signaling and allowing strong invasion abilities and high propensity to metastasize. Furthermore, the amoeboid state is associated with an immunomodulatory secretome, favoring the recruitment of protumor immune cells (mainly macrophages) within tumor microenvironment. This secretome is, in part, controlled by CD73 levels in cancer cells. Together, amoeboid pancreatic cancer cells favor invasion and metastasis in mouse models and in patients, leading to a poor prognosis. Created with BioRender.com.

Collectively, our data demonstrate that amoeboid migration is a common feature in PDAC tumors and that this cellular state is particularly important for local invasion, metastasis development, and immune evasion (Fig. 8). Therefore, strategies that aim at targeting the invasive and immunosuppressive amoeboid phenotype, such as CD73 blocking antibodies, could hold promise for this devastating disease.

## MATERIALS AND METHODS

### Study design

The objective of this study was to determine whether pancreatic cancer cells can be classified according to their morphology, contractility, and invasive features within 3D environments. We also designed experiments to study the impact of ROCK signaling within pancreatic tumors on tumor aggressiveness and metastasis, as well as a potential mechanistic link between amoeboid phenotype and immune escape. We performed experiments in this study at least with three replicates to demonstrate biological reproducibility and to ensure statistical power for comparisons. Commercially available cell lines were used for performing a range of in vitro experiments. For mouse studies, mice of similar age, sex, and size were used across all groups and were randomly assigned to each group (project license numbers PP3795393 and PBE3719B3). Tissue samples were collected from patients with PDAC who underwent surgery at University Hospital Arnau de Vilanova. All participants were well informed and signed an informed consent form. The details of study design, sample sizes (which were determined according to previous publications and experimental experience), experimental replicates, and statistics are described below, in the Supplementary Materials, and in the figure legends.

### Cell culture on thick layers of collagen I

Cells were plated on top of collagen using flowing procedure, previously described in (*21*). Bovine collagen I (no. 5005-B; PureCol, Advanced BioMatrix) thick layer was prepared at 1.7 mg/ml. After polymerization (4 hours), cells were seeded on top of collagen in medium containing 10% fetal bovine serum (FBS) and allowed to adhere for 16 hours, and the medium was changed to 1% FBS with corresponding treatments (where appropriate). Cells were analyzed 24 hours later. At the end of experiment, gels were fixed with 4% formaldehyde and imaged.

### Drug treatments

Reagents were purchased and used as follows: GSK269962A (1 μM; Axon Medchem) in dimethyl sulfoxide (DMSO; #1167, Axon) was used as a ROCKi. LY-294002 (10 μM; #1130, Tocris Bioscience) in DMSO was used as a PI3K inhibitor. Adenosine (5 μM; A4036, Sigma-Aldrich) in DMSO, APCP (10 to 100 μM; M3763, Sigma-Aldrich), and anti-CD73 antibody (clone 2C5, Medimmune/Astra-Zeneca) were used. Time of treatment and drug concentration were specified in each experiment.

### RhoA-GTP pulldown assay

A total of 250,000 cells per well were seeded on six-well plates and serum-starved (1% FBS; 24 hours). Cells were lysed in lysis buffer containing 50 mM tris (pH 7.4), 1% Triton, 10 mM MgCl_2_, 200 mM NaCl, 1 mM dithiothreitol, 0.1 mM phenylmethylsulfonyl fluoride, and EDTA-free protease inhibitor; sonicated; and spun down. A small proportion of protein lysates were separated for determination of total RhoA levels. The remaining protein lysate was incubated with glutathione *S*-transferase–conjugated with Rhotekin RBD beads for 1 hour (Cytoskeleton, #RT02). Beads were collected by centrifugation, washed, and resuspended in loading buffer. All samples were boiled for 5 min and resolved by SDS–polyacrylamide gel electrophoresis. RhoA levels were detected by immunoblot (1:1000; Cell Signaling Technology, #2117).

### Analysis of cell morphology and cellular events

Cell morphology was quantified on images of F-actin–stained cells cultured on top of bovine collagen I matrices using ImageJ. Cell morphology was assessed using the morphology descriptor tool “roundness” after manually drawing around the cell shape. Values closer to 1 represent rounded morphology; values closer to 0 represent more spread and/or spindle-shaped cells. Cellular events were quantified manually using ImageJ. Alone cells were classified as “individuals.” Groups of two cells were classified as doublets. More than two cells in contact were classified as “clusters.” Cells with membrane in contact with other cells were classified as colony. Bleb quantification was performed manually according to the presence of actin blebs with phalloidin staining.

### Microfluidic chip fabrication.

Microfluidic devices were fabricated using photolithography and soft lithography as described (*37*). A master with positive relief patterns of SU-8 2050 photoresist (A-Gas Electronic Materials) on a silicon wafer (PI-KEM) was prepared by photolithography. A polydimethylsiloxane (PDMS; Ellsworth Adhesives) polymer was cast against this master and thermally cured to obtain a negative replica piece. After separating from the master, hydrogel ports and medium reservoirs were punched from the PDMS stamp using biopsy punches. The PDMS stamp is then bonded to a glass coverslip using an oxygen plasma treatment. Devices were then autoclaved and dried at >60°C for 3 days to restore hydrophobicity. Collagen (6 mg/ml) was injected in the central channel via inlets and allowed to polymerize for 30 min, after which medium was added to the side channels. The next day, medium was aspirated, and 8000 cells were seeded in 8 μl in the top-left inlet. Chip was then flipped on the side for 30 min to allow cancer cells to enter the channel by gravity and to form adherence with collagen. Medium was then added in side inlets, and cells allowed to invade. After 24 hours, chips were fixed using 4% paraformaldehyde (PFA) and stored in phosphate-buffered saline (PBS) before immunofluorescence analysis.

### Liver metastasis on chip

The Emulate chip-S1 (Emulate Inc.) is composed of a flexible PDMS elastomer containing two parallel microchannels (1 mm by 1 mm and 1 mm by 0.2 mm for upper and lower channels, respectively) separated by a porous flexible PDMS membrane (50 μm thick, with 7-μm-diameter pores). Before cell seeding, chips were activated using Emulate’s proprietary protocols and reagents (Chip Protocols and ER solutions, Emulate Inc.). After surface functionalization, both channels of the Liver-Chip were coated with ECM (collagen I, 100 μg/ml; fibronectin, 25 μg/ml). HepG2 cells were used as hepatocyte-like cells and were seeded in the upper channel at a concentration of 3.5 million cells/ml in DMEM (10% FBS and 1% penicillin-streptomycin) and then incubated at 37°C with 5% CO_2_. LSECs were seeded at a concentration of 3 million cells/ml in CSC medium (4Z3-500, Cell Systems; 10% FBS and 1% penicillin-streptomycin) in the lower vascular channel. The next day, chips were connected to the Zoë culture module and perfused at 30 μl/hours to provide a continuous supply of fresh medium. The next day, cancer cells were mixed with buoyancy medium [1.6% (v/v) solution of Gelzan in Percoll, 50,000 cells/ml] and introduced into the lower channel as a bolus at a high flow rate of 1000 μl/hour for 4 hours. The chamber was then flushed using LSEC medium (1000 μl/hours, 30 min), and chips were imaged to confirm attachment. Both of the chip channels were perfused at 30 μl/hours to provide a continuous supply of fresh medium for the duration of the experiments. Confocal microscope (CSU-W1 SoRa Spinning disk confocal, Nikon) was used for pictures acquisition at days 0, 4, and 8 after cancer cells injection. After 8 days, chips were fixed in 4% PFA and stored in PBS before immunofluorescence analysis.

### Video microscopy cell tracking

Cells were seeded on top of a collagen I matrix, and tracking of cell migration within collagen matrices was performed over a period of 16 hours starting after the gel was set. Individual cells were tracked in a semiautomated manner by repeated random selection of cells in movie frames and manual tracking of migration pathways using the Manual Tracking plugin from ImageJ.

### Macrophage polarization

#### Human PBMC isolation

PBMCs from healthy donors were obtained from anonymized human buffy coats supplied by the NHS Blood and Transplant (Tooting, London, UK). This study was approved by the West London & GTAC Research Ethics Committee (study number 19/LO/1804). All experiments conformed to the relevant regulatory standards. Buffy coats were diluted with PBS (Gibco), and PBMC isolation was performed by Lymphoprep density gradient separation (Axis-Shield, Oslo, Norway). MACS technology was used to isolate CD14^+^ monocytes.

#### In vitro differentiation of human CD14^+^ monocytes to macrophages

A total of 2 × 10^6^ CD14^+^ monocytes per well (12-well plate) were seeded in complete RPMI and incubated in 5% CO_2_ at 37°C. On day 3, 50% of the medium was replenished with fresh medium containing conditioned media (35 μg/ml), and monocytes were incubated for three additional days. IL-4 (20 ng/ml; Peprotech, London, UK) was used as control of polarized macrophages. CD14^+^ cells with RPMI medium only served as control. On day 6, cells were collected using 2 mM EDTA on ice for flow cytometry.

#### Flow cytometry

Cells were washed once with FACS buffer (PBS^+/+^, 1% bovine serum albumin (BSA), 2 mM EDTA, and 0.1% NaN_3_) and after FcR blocking (Human TruStain FcX, BioLegend) costained for CD163-APC (1:25; clone: GHI/61; eBioscience, Hertfordshire, UK) and CD206-PE (1:25; clone: 15-2; BioLegend, London, UK). After 30 min of incubation at 4°C in the dark, cells were washed twice with FACS buffer (1500 rpm, 4°C, and 5 min) and resuspended in 500 μl of 4′,6-diamidino-2-phenylindole solution (5 μg/ml; BioLegend) for viability and immediately acquired on a LSR Fortessa III flow cytometer and analyzed using FlowJo 7.6.5 software (Tree Star). Purity of isolated CD14^+^ cells was checked by staining for CD14-PerCP-Cy5.5 (1:50; clone: HCD14; BioLegend) and was routinely >95% across all the experiments.

### Animal studies

All animals were maintained under specific pathogen–free conditions and handled in accordance with the Institutional Committees on Animal Welfare of the U.K. Home Office (The Home Office Animals Scientific Procedures Act, 1986). All animal experiments were approved by the Ethical Review Process Committees at Barts Cancer Institute, King’s College London, and The Francis Crick Institute and carried out under licenses from the Home Office, UK. All mice were obtained from Charles River UK. Mice used were 6 to 12 weeks old.

For experimental metastasis assays, intrasplenic injections were performed by surgical implantation. Briefly, cells were trypsinized, washed, and resuspended in sterile PBS. After incision on the flank of mice and extraction of the spleen, 5 × 10^5^ cells in 20 μl of PBS were injected into the spleen of C57Bl/6 female mice (KPC-claus cells) or NSG female mice (NOD/SCID/IL2Rγ^−/−^; PaTu8988T cells). Mice were anesthetized by inhalation of isoflurane, and buprenorphine (0.1 mg/kg) was used for pre- (30 min before surgery) and postoperative (4 hours after surgery) analgesia. For KPC-claus study, mice were treated daily [GSK269962A (25 mg/kg) or vehicle, 5% DMSO as control] by oral gavage. Mice were euthanized after 20 days, and then, tumors and livers were washed, fixed, and examined by immunohistochemistry. For PaTu8988T-siCD73 experimental metastasis study, mice were culled after 9 days.

### Intravital imaging studies

A total of 1 × 10^6^ PaTu8902 cells stably expressing Lifeact-GFP were suspended in 100 μl of PBS:Matrigel (50:50, v:v) and injected subcutaneously into the flank of 6- to 8-week-old CD-1 nude mice (*n* = 3). Tumor growth was monitored, and when tumors reached visible size (5 to 8 mm in diameter), mice were anesthetized and imaged as described (*71*). For intravital imaging, 7 to 10 different regions were imaged simultaneously for 2 hours for each tumor (approximately 50 μm deep on average). For motion analysis pictures, static regions appear white, whereas distinct areas of color indicate motile cells. Moving cells were defined as those that moved 10 μm or more for at least 20 min.

### Transgenic KPC model

Pdx1-Cre; LSL-Kras^G12D/+^, LSL-Trp53^R172H/+^ mice have been described previously (*38*). Mice were enrolled when tumors were detected by ultrasound monitoring (Vevo 2100; Fujifilm VisualSonics Amsterdam, NL). Enrolment was restricted to mice with tumors of a mean diameter between 4 and 10 mm. Mice were assigned to treatment group at homogeneous tumor sizes. Mice were treated (10 mg/kg, twice weekly) intraperitoneally with CD73 blocking antibody (clone 2C5) or m-IgG1 as a control. Mice were euthanized after 3 weeks of treatment or when clinical end points were met (long treatment cohort − weight loss > 20%, lethargy, unkempt appearance, and abdomen swelling), and tumors and livers were washed, fixed and examined by immunohistochemistry, or used for FACS analysis.

### Tissue microarrays

Three TMAs including formalin-fixed paraffin-embedded (FFPE) biopsies of 44 human pancreatic adenocarcinomas were included in the cohort A. Each biopsy was represented by two or three cores (diameter, 1 mm). Survival data were available for 40 patients. Clinical stage data were available for 39 patients. Tumor samples for cohort A were processed by IRBLleida (PT17/0015/0027) and HUB-ICO-IDIBELL (PT17/0015/0024) Biobanks integrated in the Spanish National Biobank Network and Xarxa de Bancs de Tumors de Catalunya following standard operating procedures with the appropriate approval of the Ethics and Scientific Committee. Samples were collected with specific informed consent, in accordance with the Helsinki Declaration. TMA of the cohort B were obtained from H. Kocher (Barts Cancer Institute, London, UK). Tissue microarrays in cohort B were constructed with pancreatic tissues collected at cancer resection or biopsy at Barts Health NHS Trust [City and East London Research Ethics Committee (REC) 07/0705/87].

### Immunohistochemistry

#### Experimental procedure

All tissue samples were FFPE, sectioned (3 to 4 μm thick), and dried for 1 hour at 65°C. Next, tissue samples were subjected to deparaffinization, rehydration, and heat-induced epitope retrieval using a Biocare Decloaking Chamber (DC2012) at 110°C for 7 min in the corresponding unmasking solution. Sections were then incubated with primary antibody overnight at 4°C, washed, and HRP-coupled secondary antibody for 30 min. Reagents were incubated at room temperature in a humidified slide chamber. Revelation was performed using ImmPACT VIP Substrate (SK-4605, Vectorlabs). All the stainings were counterstained with hematoxylin. For Masson’s trichrome, staining was performed according to manufacturer’s recommendations (ab150686, Abcam).

#### Digital pathology: Imaging and scoring

Whole-section images were scanned using NanoZoomer S210 slide scanner (Hamamatsu). For liver metastasis quantification, metastatic areas were manually drawn using NDP.View.2 software (Hamamatsu). For KPC pMLC2 (CST#3671) and CD73 (CST#13160) stainings, quantification was performed with QuPath software. Positive cell detection was performed with a defined threshold. Next, software was trained creating random trees classification algorithm combined with the intensity information to morphologically differentiate tumor cells from necrosis, stroma, and immune cells. TB staining was quantified at the center of the tumor mass. IF staining was quantified within a 400-μm-width area at the border of the tumor mass.

For amoeboid scoring, sections from the TMA were stained using CK-19 antibody (ab9221) to identify cancer cells. Amoeboid cells were defined as individual, round, CK-19–positive cells, and tumor sections were scored according to the absence or the presence (at least one amoeboid cell on the whole section) of amoeboid cells. Patients were then sorted as “amoeboid positive” or “amoeboid negative” according to the most frequent score of the biopsies’ sections. For CAF pMLC2 scoring, sections from the TMA were stained using pMLC2 (CST#3671) antibody. A score from 0 (no pMLC2 staining in CAF) to 2 (strong pMLC2 staining in CAF) was given to each section, and sections with a score of 1 and 2 were defined as “high fibroblastic pMLC2.” For CD73 stainings (CST#13160), quantification was performed with QuPath software. Positive cell detection was performed with a defined threshold. Next, software was trained creating random trees classification algorithm combined with the intensity information to morphologically differentiate tumor cells from necrosis, stroma, and immune cells. High CD73 tumors were defined as tumors with an average of ≥10% of CD73-positive cancer cells.

For α–smooth muscle actin (ab5694, Abcam), CD68 (ab74704, Abcam), and Masson’s trichrome stainings, quantification was performed with QuPath software. Positive areas were detected with a defined threshold. For CD163 stainings (MCA1853, Bio-Rad), quantification was performed with QuPath software. Positive cells were detected with a defined threshold.

Pseudo-colored digital multiplex images were generated as previously described (*25*, *72*). Briefly, images were aligned, and colors were deconvoluted using ImageJ software. Merge images were generated using ImageJ by overlaying pseudo-color images for each staining. Images fluorescence were then separated and analyzed using QuPath.

### Statistical analysis

Student’s test, Mann-Whitney’s test, one-way and two-way analysis of variance (ANOVA) with Tukey or Sidak’s post hoc test, Kruskal-Wallis with Dunn’s multiple comparison test, and survival curve estimation based on the Kaplan-Meier method and log-rank test were performed using GraphPad Prism (GraphPad Software Inc.). Data were plotted as graphs showing mean ± SEM as indicated in the figure legends. *P* values were calculated using two-sided tests. *P* values of less than 0.05 were considered statistically significant. In the figure legends, “*n*” means number of independent experiments unless otherwise stated (*P < 0.05, **P < 0.01, ***P < 0.001, and ****P < 0.0001).

## Supplementary Materials

### This PDF file includes:

Supplementary Text Figs. S1 to S7 Legends for movies S1 to S5 References
