# Supplementary material for: CD73 controls Myosin II–driven invasion, metastasis, and immunosuppression in amoeboid pancreatic cancer cells
Source: Sci Adv. 2023 Oct 18;9(42):eadi0244. doi: 10.1126/sciadv.adi0244 (PMC10584351; doi:10.1126/sciadv.adi0244)
Supplement: Supplementary file 1 — Supplementary Text Figs. S1 to S7 Legends for movies S1 to S5 References [file sciadv.adi0244_sm.pdf]

Science Advances
AAAS

Supplementary Materials for

CD73 controls Myosin II–driven invasion, metastasis, and immunosuppression in amoeboid pancreatic cancer cells

Remi Samain *et al.*

Corresponding author: Victoria Sanz-Moreno, v.sanz-moreno@qmul.ac.uk

Sci. Adv. **9**, eadi0244 (2023)
DOI: 10.1126/sciadv.adi0244

The PDF file includes:

Supplementary Text
Figs. S1 to S7
Legends for movies S1 to S5
References

Other Supplementary Material for this manuscript includes the following:

Movies S1 to S5

CD73 controls amoeboid invasion and immunosuppression in PDAC

SUPPLEMENTARY MATERIALS

Supplementary methods

Cell culture

Cells were grown at 37ºC and 5% CO2 in DMEM supplemented with 10% FBS and 1% penicillin/streptomycin (all from Gibco). Panc1, PaTu8988T and Cfpac1 cell lines were a kind gift from Professor Tatjana Crnogorac-Jurcevic (Barts Cancer Institute, London, UK); SW1990, Colo357, Capan2 and Patu8902 cell lines were from Doctor Claire Wells (King's College, London, UK), and PaTu8988S cell line was purchased from the German Collection of Microorganisms and Cell Cultures (DSMZ, Leibniz Institute, Germany). KPC-claus cells were from Doctor Claus Jorgensen's lab. All cell lines were regularly tested for mycoplasma contamination and cell phenotypes were verified routinely.

Transfection and siRNA

250K cells/well were seeded on 6-well plates and transfected with 20nM SmartPool siRNA oligonucleotides, using Optimem-I and Lipofectamine 2000 (Invitrogen). Non-targeting siRNA was used as control. Transfected cells were incubated for 24 h, after which they were harvested and re-seeded for conditioned media or western-blot experiments.

Immunoblotting and antibodies

Cells on plastic were lysed in Laemmli sample buffer, boiled, sonicated and spun down. Lysates were fractionated using 8%, 10% or 12% SDS-polyacrylamide gel electrophoresis and transferred onto PVDF filters (0.45 µm, Immobilon). ECL or Prime ECL detection Systems (GE Healthcare) with HRP-conjugated secondary antibodies (GE Healthcare) were used for detection. Antibodies: pThr18/Ser19-MLC2 (#3674, 1:750), MLC2 (#3672, 1:750), E-cadherin (#3195, 1:1000), Vimentin (#5741, 1:1000), β-Catenin (#8480, 1:1000) and Snail (#3879, 1:1000), CD13 (#32720, 1:1000) pSer473-Akt (#9271), Akt (#9272), CD73 (#13160, 1:1000) from Cell Signalling Technology; Cytokeratin-19 (ab9221, 1:1000), CD44 (ab157107, 1:5000), Nanog (ab80892, 1:500), Oct4 (ab181557, 1:1000) from Abcam; KLF4 (NBP1-83940, 1:1000) from Novus Biologicals; GAPDH (MAB374, 1:5000) from Millipore.

Confocal fluorescence microscopy and image quantification

Cells were seeded on top of a collagen I matrix and immunostained as described. Cells were fixed with 4% formaldehyde, permeabilised with 0.2% Triton X-100, blocked with 5% bovine serum albumin (BSA), stained with primary antibodies pSer19-MLC2 (#3671, 1:200) and E-cadherin (#3195, 1:200) from Cell Signalling and CD44 (ab157107, 1/5000) from Abcam, which were detected with secondary Alexa Fluor 488 or 647 antibodies (Life Technologies). F-actin was stained using Alexa Fluor 647-phalloidin (Life Technologies) and DNA with Hoechst 33258 (Life Technologies). Imaging was carried out on a Zeiss LSM 510 Meta confocal microscope (Carl Zeiss) with C-Apochromat × 40/1.2 NA (water) or a Plan Apochromat × 63/1.4 NA (oil) objective lenses and Zen software (Carl Zeiss). p-MLC2 fluorescence signal was quantified calculating the pixel intensity in single cells relative to the cell area.

3D invasion assays

Experiment was performed as described in *(21)*. Briefly, cells were suspended in serum-free bovine collagen I at 2.3 mg/ml to a final concentration of $1,5x10^4$ cells per 100 µl of matrix, seeded on 96-wel*(21)*l plates and spun down to the bottom of the well. After the matrix was polymerized, 10% FBS-containing media was added on top of the matrix, allowing the cells to invade upwards for 24 h as previously described. Then plates were fixed with 4% formaldehyde, stained with 5 µg/ml Hoechst 33258 (Life Technologies) and imaged on a Zeiss LSM 510 Meta confocal microscope (Carl Zeiss) with Zen software. Confocal z-slices were collected from each well at the bottom of the well and at 50µm. The 3D migration index was calculated as number of invading cells at 50 µm divided by the total number of cells at the bottom.

Spheroid invasion assays

Cells were resuspended in 1ml of low viscosity medium (800 µL of DMEM 10% FBS with 200 µL of low viscosity methylcellulose solution) at a final concentration of $4x10^5$ cells/ml. 40 droplets (25 µL each) were plated on the lid of a tissue culture dish for 48 hours at 37ºC – 5% CO2, allowing cells to cluster into compact spheroids. Then, spheroids were collected, centrifuged (300 rpm, 15 seconds), and resuspended in a collagen I solution (600µl, 1.7mg/mL in DMEM). Spheroids were seeded in 2 wells of a 24-well plates (300µl each) and left in the incubator at 37ºC – 5% CO2 for 4 hours to allow matrix polymerization. Medium with 10% FBS (supplemented with treatments if needed) was added on top and phase contrast pictures were taken every 24 hours, during 4 days. For spheroid invasion quantification, the increase on the area occupied by the spheroids between day 0 (when spheroids were embedded into the collagen I matrix) and day 4 was calculated by using ImageJ software. If needed, plates were

fixed with 4% formaldehyde, stained with Hoechst 33258 (Life Technologies) and Alexa Fluor 647-phalloidin (Life Technologies) and imaged on a Zeiss LSM 510 Meta confocal microscope (Carl Zeiss) with Zen software.

Cytokine array

Secreted media from PaTu8988T, PaTu8988S or PaTu8988T transfected with a non-targeting or a *NT5E*-targeting siRNA were collected after 48h in serum-free conditions and incubated with Human Cytokine Antibody Array (RayBiotech, Inc., C4000), following the manufacturer's protocol. Membranes were incubated with biotinylated detection antibody cocktail, with HRP-conjugated streptavidin and with detection buffers. Images were obtained using a chemoluminescent imaging system and densitometry analysis was performed using the Protein Array Analyser plugin for ImageJ (http://image.bio.methods.free.fr/ImageJ/?Protein-Array-Analyzer-for-ImageJ.html).

Gene set enrichment analyses (GSEA)

Normalized gene expression microarray data of epithelial versus mesenchymal PDAC cells was analysed by comparing PaTu8988T and PaTu8988S cell lines (GSE14701 *(32)*). Normalized gene expression microarray data of amoeboid melanoma cells were analysed by comparing amoeboid A375M2 cells to A375M2 cells treated with ROCK inhibitors (H1152 and Y27632) or blebbistatin (GSE23764 *(29)*). Normalized gene expression microarray data of tumoral pancreas were analysed by comparing adjacent and normal pancreatic tissue to tumour tissue (GSE71729 *(51)*; GSE28735 *(73)*). Gene sets for epithelial-to-mesenchymal transition, tumour zone peripheral versus central signature, metastasis signature and pancreatic cancer gene signature were

downloaded and analysed using Gene Set Enrichment Analysis (GSEA) software (http://www.broadinstitute.org/gsea/index.jsp). For the transcriptional signature of melanoma cells with high myosin II activity, genes upregulated in high myosin II activity compared to low myosin II activity melanoma cells (cells treated with ROCKi and blebbistatin) were selected using a fold change >1.5 and a p value <0.01. GSEA analysis was performed as described above. Enrichment plot (green line) show upregulation of gene signature in indicated samples (resistant, non-responders or on-treatment). Analysis was performed with the specific settings: permutations-1,000, permutation type-gene set, metric for ranking genes-t-test.

Enrichment analysis

Enrichment networks and process networks were obtained using the ClueGO plug-in from Cytoscape software *(74)* (http://www.ici.upmc.fr/cluego/cluegoDownload.shtml). Biological processes enriched in gene lists were identified using g:Profiler web server *(75)*.

Analysis of gene expression data and immunostainings from human databases

Gene expression data, clinical information and Cytokeration-19 IHC pictures of human pancreatic cancer samples (176 patients) from The Cancer Genome Atlas (TCGA) database were downloaded from The Human Protein Atlas website (https://www.proteinatlas.org/). Comparison of gene expression between normal and tumoural pancreas tissue from TCGA, as well as correlation between genes from TCGA, were performed using OncoDB website *(76)* (https://oncodb.org/index.html). Single cell data were obtained from the Tumor Immune Single-cell Hub 2 (TISCH2) database (*http://tisch.comp-genomics.org/home/)(77)*.

Immune cell distribution estimation using CIBERSORT

Normalized gene expression data of tumoral pancreas (GSE71729 *(51)*) were used for immune cell proportion estimation. Samples were sorted according to *ROCK1* and *NT5E* median expression and their RNA signatures were analysed using CIBERSORT analytical tool (https://cibersort.stanford.edu/), using LM22 signature gene file (100 permutations for statistical analysis).

FACS analysis of KPC Tumours

Pancreas was collected in ice-cold PBS and washed in Hank's balanced salt solution (HBSS) solution before mincing using scalpels. The pieces were then incubated in 2 mg/mL collagenase (Sigma) in HBSS with 50 mg/mL DNase (Sigma) for 20 min at 37°C in a shaker. The pieces were then passed through a 70-mm cell strainer and resuspended in flow cytometry buffer and cells counted. Antibody labelling and flow cytometry were carried out using standard protocols. Cells were plated in a 96-well plate (3 million cells/well) and resuspended in Fc block (CD16/32, eBioscience), incubated on ice and mastermix of antibodies added. After washing cells were stained with a fixable viability dye-e506 (eBioscience) in PBS. Cells were then resuspended in Fluorophix (Biolegend) and cells were washed and resuspended in flow cytometry buffer. Flow cytometric analysis was performed using an LSRFortessa cell analyzer (BD Biosciences) and FACSDiva software Version 6.2. Data were transferred and analyzed using the FlowJo software (Tree Star, Oregon, USA). Antibodies used: CD45 (30-F11), CD3 (145-2C11), CD4 (RM4-5), CD8 (53-6.7), CD11b (M1/70), F4/80 (BM8), all from Biolegend. Ly6G/Gr1 (RB6-8C5) antibody was from eBioscience.

Supplementary Figures

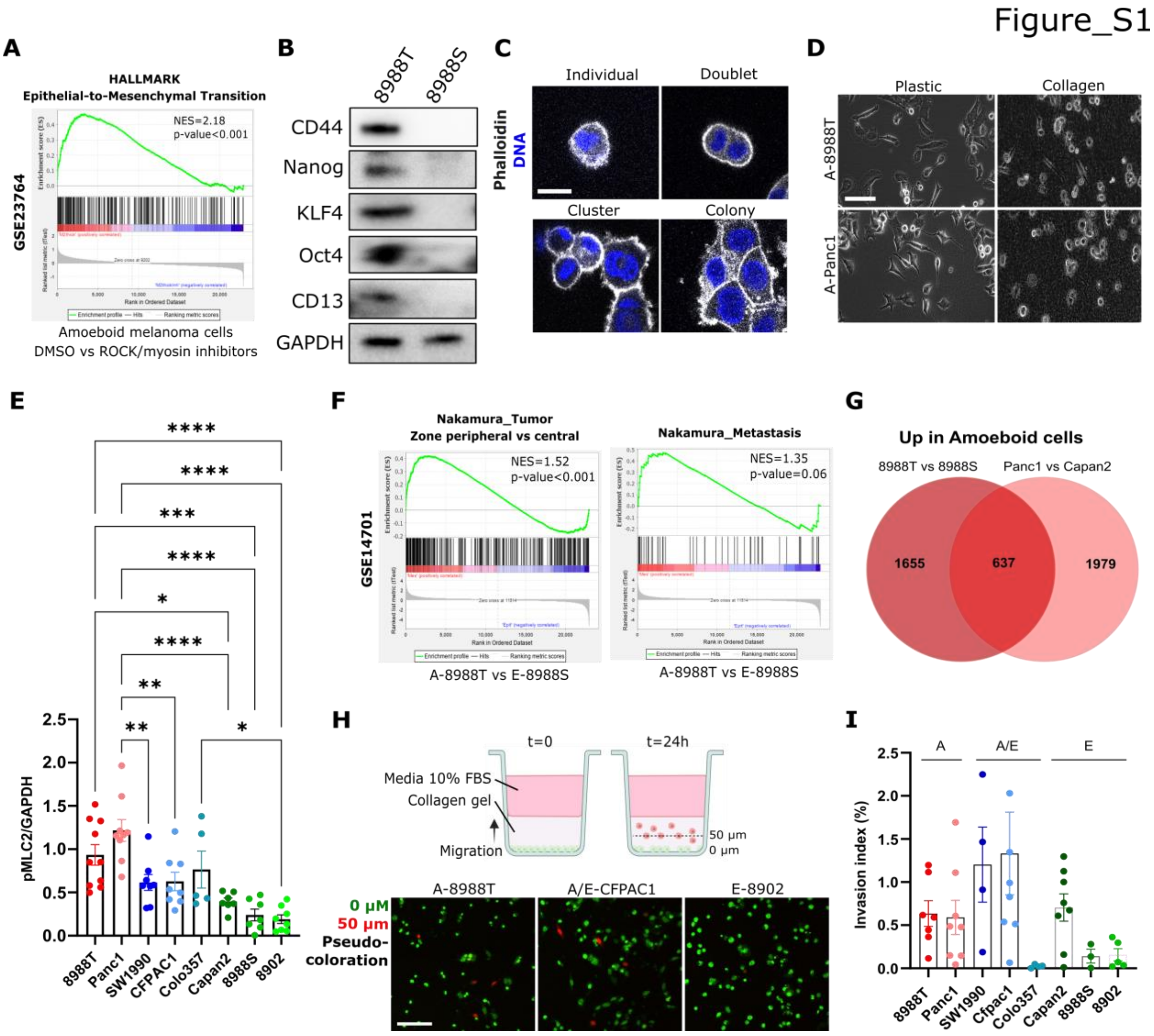

Supplementary Figure 1. Related to Figure 1.

A, GSEA plots showing enrichment of “epithelial-to-mesenchymal” gene signature in melanoma amoeboid cells compared to amoeboid cells treated with ROCK and myosin inhibitors. **B,** Representative immuno-blots of CD44, Nanog, KLF4, Oct4, CD13 and GAPDH for PaTu8988T and PaTu8988S cells. **C,** F-actin confocal images of individual, doublet, cluster and colony-forming cells. Scale bar 10 µm. **D,** Representative bright field pictures of PaTu8988T and Panc1 cells plated on plastic or on top of a collagen gel. Scale bar 25 µm. **E,** Quantification of normalized pMLC2 levels from (1F) immunoblots (n=5 to 10). **F,** GSEA plots showing enrichment of “tumour periphery” and “metastasis” gene signatures in PaTu8988T

cells compared to PaTu8988S cells. **G,** Venn diagram showing genes upregulated in PaTu8988T cells compared to PaTu8988S cells, in Panc1 cells compared to Capan2 cells, and commonly upregulated. **H,** Schematic of the invasion assay protocol (upper panel); representative merged images of PaTu8988T, Cfpac1 and PaTu8988S cells at the bottom (green) and invading at 50 µm (red). Scale bar 100 µm (lower panel). **I,** Quantification of 3D invasion index of PDAC cell lines (n≥3, normalized to PaTu8988T cell line, each dot represents the average index in an independent experiment). (E, I) graphs show mean +/- SEM. P-values to compare pMLC2 levels (E) were calculated using one-way ANOVA with Tukey's multiple comparison tests.

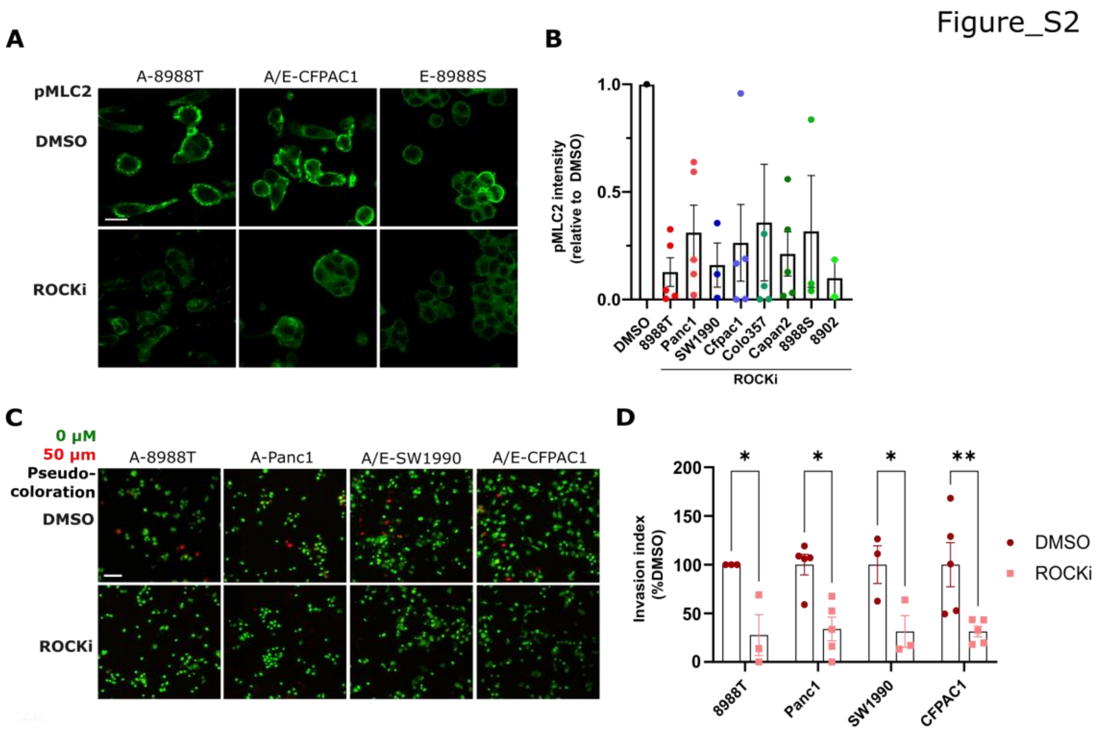

Supplementary Figure 2. Related to Figure 2.

A, pMLC2 confocal images in PaTu8988T, Cfpac1 and PaTu8988S cells treated with DMSO (control) or 1 µM GSK269962A for 24 hours. Scale bar 10 µm. **B,** Quantification of pMLC2 immunofluorescence signal normalized by cell area (n≥3, intensity relative to the same cell line treated with DMSO, each value represents average intensity in an independent experiment). **C,** Representative merged images of PaTu8988T, Panc1, SW1990 and Cfpac1

cell lines treated with 1 µM GSK269962A or DMSO (control) for 24 hours at the bottom (green) and invading at 50 µm (red). Scale bar 50 µm. **D,** Quantification of 3D invasion index of A and A/E PDAC cell lines treated with 1 µM GSK269962A or DMSO (control) for 24 hours ($n \geq 3$, normalized to DMSO index, each dot represents the average invasion index in an independent experiment). (B,D) graphs show mean +/- SEM. P-values to compare invasion index (D) were calculated using two-way ANOVA with Sidak's multiple comparison test.

Figure_S3

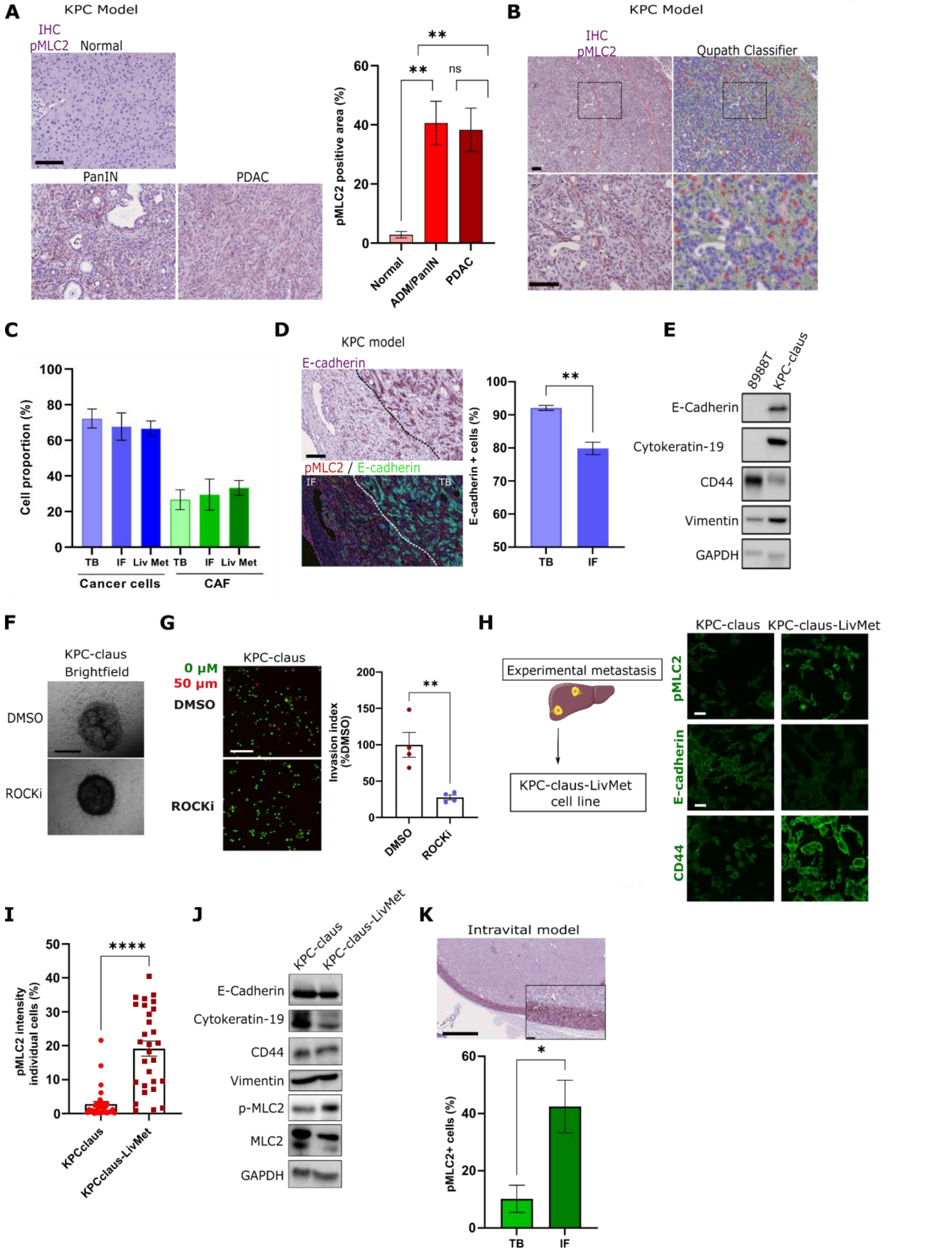

A
KPC Model
IHC pMLC2
Normal
PanIN
PDAC
pMLC2 positive area (%)
Normal
ADM/PanIN
PDAC
**
ns
B
KPC Model
IHC pMLC2
Qupath Classifier
C
Cell proportion (%)
TB
IF
Liv Met
Cancer cells
CAF
D
KPC model
E-cadherin
pMLC2 / E-cadherin
IF
TB
E-cadherin + cells (%)
E
8988T
KPC-claus
E-Cadherin
Cytokeratin-19
CD44
Vimentin
GAPDH
F
KPC-claus Brightfield
DMSO
ROCKi
G
0 µM
50 µm
KPC-claus
DMSO
ROCKi
Invasion index (%DMSO)
H
Experimental metastasis
KPC-claus-LivMet cell line
KPC-claus
KPC-claus-LivMet
pMLC2
E-cadherin
CD44
I
pMLC2 intensity individual cells (%)
****
KPCclaus
KPCclaus-LivMet
J
KPC-claus
KPC-claus-LivMet
E-Cadherin
Cytokeratin-19
CD44
Vimentin
p-MLC2
MLC2
GAPDH
K
Intravital model
pMLC2+ cells (%)
*
TB
IF

Supplementary Figure 3. Related to Figure 3.

A, Representative pMLC2 immunostainings of KPC mouse normal pancreas, PanIN and PDAC (left panel, scale bar 100 µm) and quantification of the proportion of pMLC2 positive cells within each compartment (right panel, n≥7). **B,** Representative pMLC2 immunostainings of KPC mouse tumours and corresponding Qupath map classifier, showing cancer cells (blue), fibroblasts (green), immune cells (purple) and pMLC2-positive cells (red). Scale bar 100 µm. **C,** Quantification of the proportion of cancer cells and cancer-associated fibroblasts (CAF) within KPC tumour bulk, invasion front and liver metastasis (n≥4). **D**, Representative E-cadherin and E-cadherin/pMLC2 pseudo-coloured multiplex images of KPC tumour bulk (TB) and invasion front (IF) (left panel, scale bar 100 µm) and quantification of the proportion of E-cadherin positive cancer cells within KPC tumour bulk and invasion front (right panel, n≥5). **E,** Representative immuno-blots of E-cadherin, Cytokeratin-19, CD44, Vimentin and GAPDH of PaTu8988T and KPC-claus cells. **F,** Representative bright field (left panel, scale bar 500 µm) images of KPC-claus spheroids treated with 1 µM GSK269962A or DMSO (control) at day 4. **G,** Representative merged images of KPC-claus cells treated with 1 µM GSK269962A or DMSO (control) for 24 hours at the bottom (green) and invading at 50 µm (red) (left panel, scale bar 100 µm), and quantification of 3D invasion index of KPC-claus cells treated with 1 µM GSK269962A or DMSO (control) for 24 hours (right panel, n=4 technical replicate, normalized to DMSO index). **H,** Origin of KPC-claus-LivMet cells (left panel); pMLC2, E-cadherin and CD44 confocal immunofluorescence images (right panel, scale bar 40 µm). **I,** quantification of pMLC2 immunofluorescence signal normalized by cell area (right panel, n≥30, each dot represents a cell). **J,** Representative immuno-blots of E-cadherin, Cytokeratin-19, CD44, Vimentin, pMLC2, total MLC2 and GAPDH of KPC-claus and KPC-claus-LivMet cells. **K,** Representative pMLC2 immunostaining of an intravital PaTu8902-GFP tumour (upper panel, scale bar 1mm; inset scale bar 100 µm) and quantification of the proportion of pMLC2 positive cells within tumour bulk and invasion front (lower panel, n=3). (A, C, D, G, I, K) graphs show mean +/- SEM. P-values to compare percentage of pMLC2 positive cells (B) were

calculated using one-way ANOVA with Tukey's multiple comparison test. P-values to compare E-cadherin positive cells (D) and invasion index (G) were calculated using Student's t-test. P-value to compare p-MLC2 intensities (I) was calculated using Mann–Whitney test. P-value to compare percentage of pMLC2 positive cells (K) was calculated using paired Student's t-test.

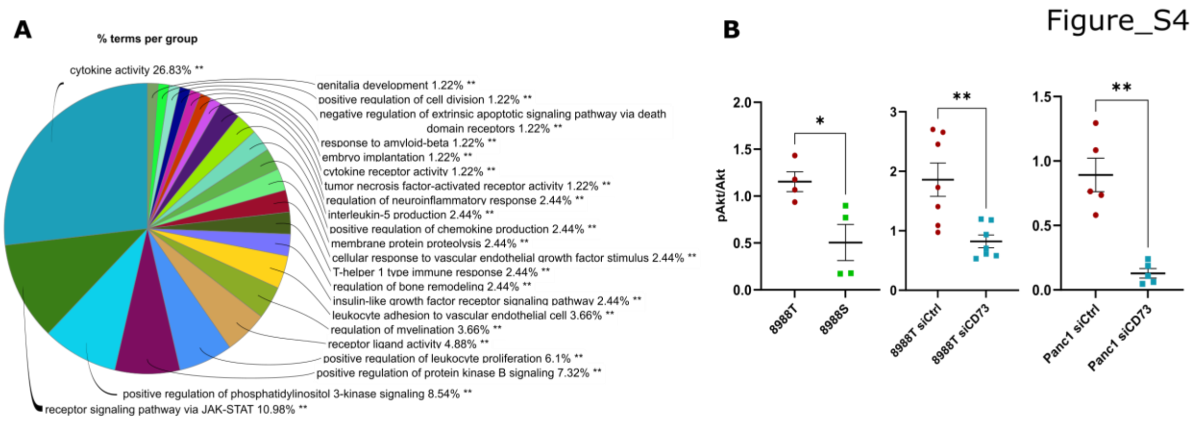

Supplementary Figure 4. Related to Figure 4.

A, Pie chart showing biological process enriched with secreted factors upregulated in PaTu8988T cells. **B,** Quantification of normalized p-Akt levels from (4H) immunoblots (n=4 to 7). (B) graphs show mean +/- SEM. P-values to compare p-Akt levels were calculated using Student's t-tests.

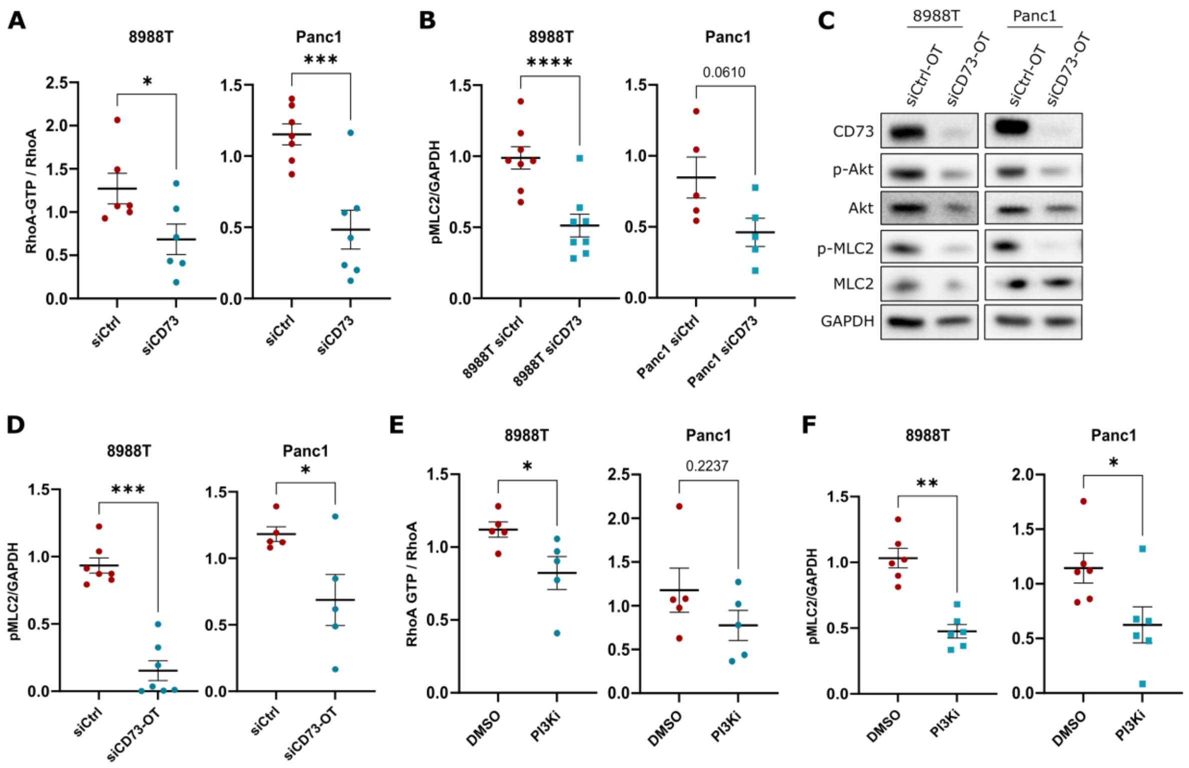

Supplementary Figure 5. Related to Figure 5.

A, Quantification of normalized RhoA-GTP levels from (5C) pulldowns (n=6 to 7). **B,** Quantification of normalized p-MLC2 levels from (5D) immunoblots (n=5 to 8). **C,** Representative immuno-blots of CD73, p-Akt, Akt, p-MLC2, MLC2 and GAPDH in PaTu8988T and Panc1 cells transfected with a control or a *NT5E* On-target siRNA. **D,** Quantification of normalized p-MLC2 levels from (S5C) immunoblots (n=5 to 7). **E,** Quantification of normalized RhoA-GTP levels from (5E) pulldowns (n=5). **F,** Quantification of normalized p-MLC2 levels from (5F) immunoblots (n=6). (A, B, D, E, F) graphs show mean +/- SEM. P-values to compare RhoA-GTP levels and p-MLC2 levels (A, B, D, E, F) were calculated using Student's t-test.

Figure_S6

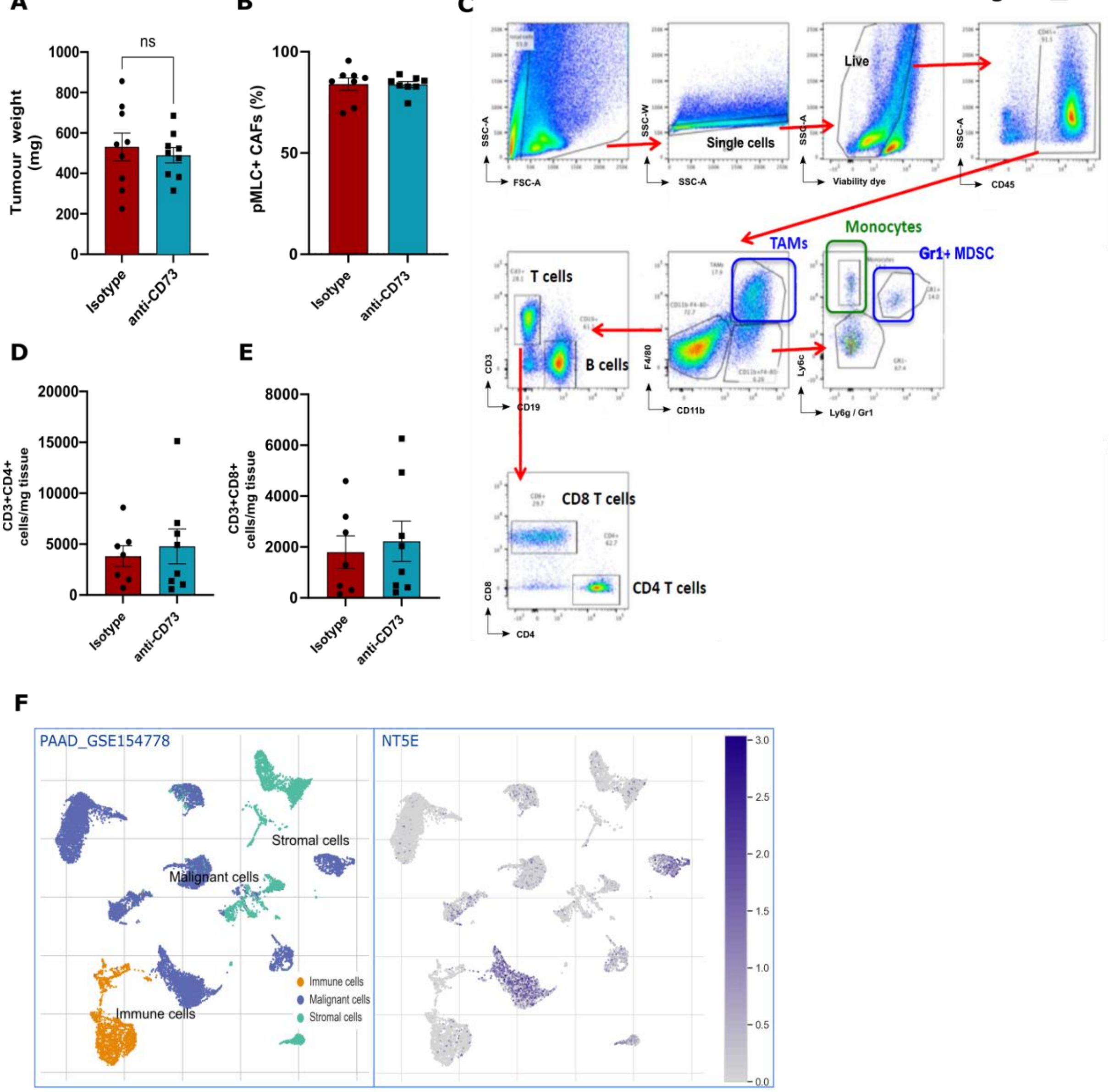

Supplementary Figure 6. Related to Figure 6.

A, Weights of KPC tumours after 2 weeks of treatment with control isotype (n=9 mice) or anti-CD73 (n=9 mice). **B,** Quantification of the proportion of pMLC2-positive cancer-associated fibroblasts (CAFs) within KPC tumours after treatment with control isotype (n=9 mice) or anti-CD73 (n=8 mice) (mice with no PDAC stage tumours were excluded from the analysis). **C,** Gating strategy for KPC tumours FACS analysis. **D,** FACS analysis of CD3+CD4+ lymphocytes per mg of KPC tumour tissue after treatment with control isotype (n=7 mice) or anti-CD73 (n=8 mice). **E,** FACS analysis of CD3+CD8+ T lymphocytes per mg of KPC tumour tissue after treatment with control isotype (n=7 mice) or anti-CD73 (n=8 mice). **F, (Left)**

UMAP plot showing the distinct cellular clusters annotated and colour-coded from 14953 cells of primary PDAC tumours (n=16 patient samples, from GSE154778), (**right**) NT5E activity score enriched in some malignant cell clusters. (A, B, D, E) graphs show mean +/- SEM.

Figure_S7

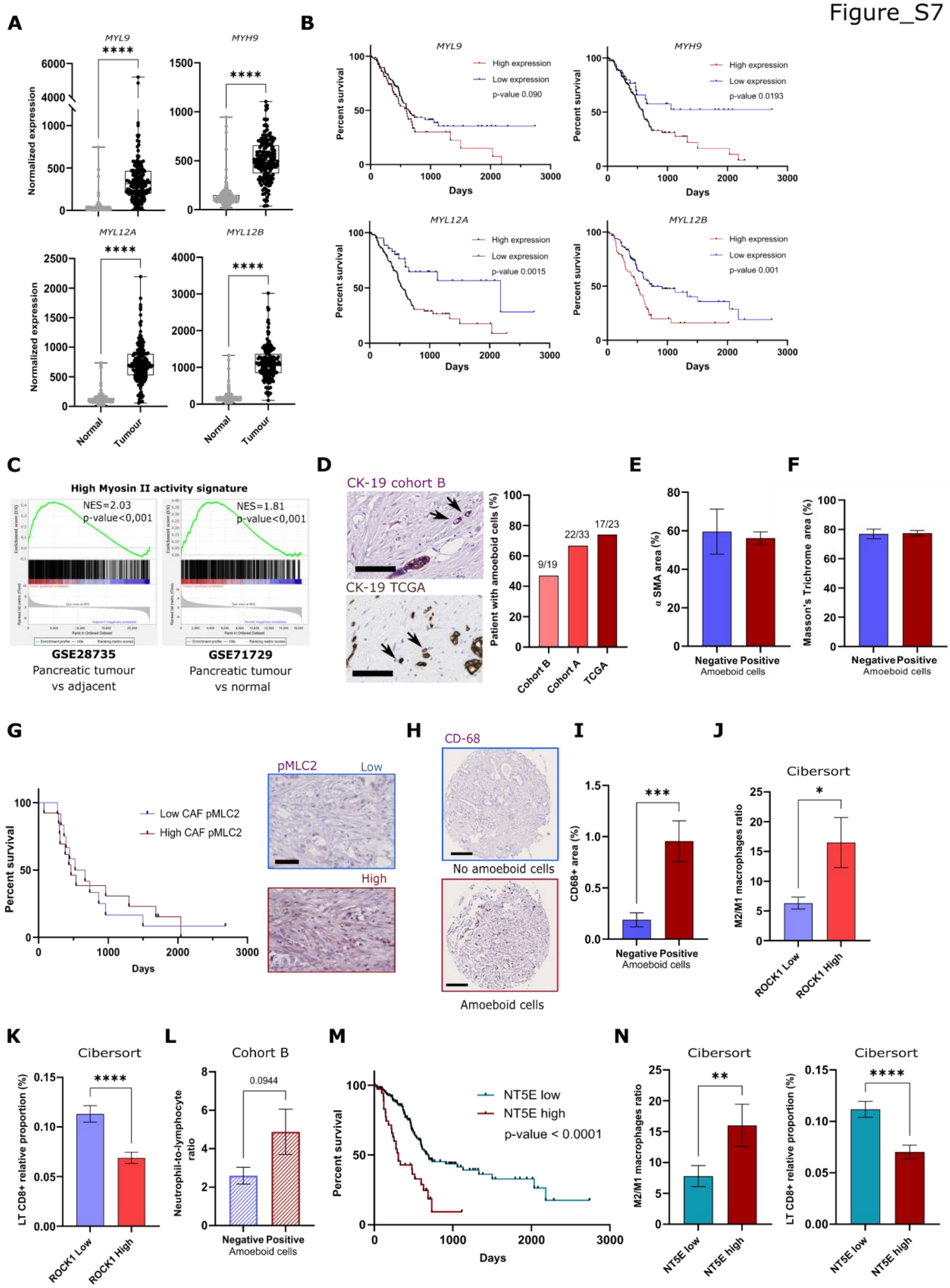

A
MYL9
MYH9
MYL12A
MYL12B
Normalized expression
Normal
Tumour
B
Percent survival
Days
High expression
Low expression
p-value 0.090
p-value 0.0193
p-value 0.0015
p-value 0.001
C
High Myosin II activity signature
NES=2.03
p-value<0,001
NES=1.81
p-value<0,001
GSE28735
Pancreatic tumour vs adjacent
GSE71729
Pancreatic tumour vs normal
D
CK-19 cohort B
CK-19 TCGA
Patient with amoeboid cells (%)
9/19
22/33
17/23
Cohort B
Cohort A
TCGA
E
α SMA area (%)
Negative Positive
Amoeboid cells
F
Masson's Trichrome area (%)
Negative Positive
Amoeboid cells
G
Low CAF pMLC2
High CAF pMLC2
Percent survival
Days
pMLC2
Low
High
H
CD-68
No amoeboid cells
Amoeboid cells
I
CD68+ area (%)
Negative Positive
Amoeboid cells
J
Cibersort
M2/M1 macrophages ratio
ROCK1 Low
ROCK1 High
K
Cibersort
LT CD8+ relative proportion (%)
ROCK1 Low
ROCK1 High
L
Cohort B
Neutrophil-to-lymphocyte ratio
0.0944
Negative Positive
Amoeboid cells
M
NT5E low
NT5E high
p-value < 0.0001
Percent survival
Days
N
Cibersort
M2/M1 macrophages ratio
LT CD8+ relative proportion (%)
NT5E low
NT5E high

Supplementary Figure 7. Related to Figure 7.

A, Normalized mRNA gene expression of *MYL9, MYH9, MYL12A and MYL12B* in normal (n=200) and tumoural (n=176) pancreas of patients from TCGA research network. **B,** Kaplan–Meier survival plot of 176 patients sorted according to gene expressions of *MYL9*, *MYH9*, *MYL12A* and *MYL12B* from TCGA research network. **C,** GSEA plots showing enrichment of "High myosin II activity" gene signature in pancreatic cancer compared to adjacent-normal pancreas. **D,** Representative pictures of cytokeratin-positive round individual cells from the cohort B (upper panel) and from the TCGA database (lower panel) (left, scale bar 100 µm), and representation of the percentage of patients with observable amoeboid cells in the 3 cohorts (right). **E, F,** Quantification of the αSMA and Masson's trichrome positive areas in amoeboid negative and positive tumour sections. **G,** Kaplan–Meier survival plot of cohort A patients sorted according to intensity score of pMLC2 staining in cancer-associated fibroblasts (right panel, scale bar 50 µm). **H,** Representative CD68 immunostainings of amoeboid negative and positive tumours from TMA sections of human PDAC. Scale bar 250 µm. **I,** Quantification of the average CD68 positive area in amoeboid negative and positive sections (n=47-106). **J,** CIBERSORT estimations of the M2/M1 macrophage ratio in *ROCK1* low (n=72) and high (n=73) tumours from GSE71729. **K,** CIBERSORT estimations of the CD8+ T lymphocytes proportions (relative to all immune cells) in *ROCK1* low (n=72) and high (n=73) tumours from GSE71729. **L,** Neutrophils-to-lymphocytes ratio in amoeboid negative and positive patients from the cohort B (n=8-10). **M,** Kaplan–Meier survival plot of 176 patients sorted according to gene expressions of *NT5E* from TCGA research network. **N,** CIBERSORT estimations of the M2/M1 macrophage ratio and CD8+ T lymphocytes in *NT5E* low (n=72) and high (n=73) tumours from GSE71729. (A, E, F, I, J, K, L, N) graphs show mean +/- SEM. P-values to compare gene expressions (A), CD68+ area (I), M2/M1 ratio (J) and CD8 lymphocytes proportion (K) were calculated using Mann–Whitney test. P-values to compare survival (G, M) were calculated using log-rank Mantel-Cox tests. P-value to compare NLR (L) was calculated using Student's t- test with Welch's correction.

Supplementary movies

Supplementary movie 1: Time-lapse Patu8988T-DMSO

Supplementary movie 2: Time-lapse Patu8988T-ROCKi

Supplementary movie 3: Time-lapse Patu8988S-DMSO

Supplementary movie 4: Intravital-invasive front

Supplementary movie 5: Intravital-tumour body

REFERENCES AND NOTES

1. L. Rahib, B. D. Smith, R. Aizenberg, A. B. Rosenzweig, J. M. Fleshman, L. M. Matrisian, Projecting cancer incidence and deaths to 2030: The unexpected burden of thyroid, liver, and pancreas cancers in the United States. *Cancer Res.* **74**, 2913–2921 (2014).

2. J. Kleeff, M. Korc, M. Apte, C. La Vecchia, C. D. Johnson, A. V. Biankin, R. E. Neale, M. Tempero, D. A. Tuveson, R. H. Hruban, J. P. Neoptolemos, Pancreatic cancer, *Nat. Rev. Dis. Primer* **2**, 16022 (2016).

3. C. L. Chaffer, R. A. Weinberg, A perspective on cancer cell metastasis. *Science* **331**, 1559–1564 (2011).

4. P. Friedl, S. Alexander, Cancer invasion and the microenvironment: Plasticity and reciprocity. *Cell* **147**, 992–1009 (2011).

5. A. G. Clark, D. M. Vignjevic, Modes of cancer cell invasion and the role of the microenvironment. *Curr. Opin. Cell Biol.* **36**, 13–22 (2015).

6. E. Sahai, I. Astsaturov, E. Cukierman, D. G. DeNardo, M. Egeblad, R. M. Evans, D. Fearon, F. R. Greten, S. R. Hingorani, T. Hunter, R. O. Hynes, R. K. Jain, T. Janowitz, C. Jorgensen, A. C. Kimmelman, M. G. Kolonin, R. G. Maki, R. S. Powers, E. Puré, D. C. Ramirez, R. Scherz-Shouval, M. H. Sherman, S. Stewart, T. D. Tlsty, D. A. Tuveson, F. M. Watt, V. Weaver, A. T. Weeraratna, Z. Werb, A framework for advancing our understanding of cancer-associated fibroblasts. *Nat. Rev. Cancer*, **20**, 174–186 (2020).

7. P. Pandya, J. L. Orgaz, V. Sanz-Moreno, Modes of invasion during tumour dissemination. *Mol. Oncol.* **11**, 5–27 (2017).

8. V. Graziani, I. Rodriguez-Hernandez, O. Maiques, V. Sanz-Moreno, The amoeboid state as part of the epithelial-to-mesenchymal transition programme. *Trends Cell Biol.* **32**, 228–242 (2022).

9. A. J. Lomakin, C. J. Cattin, D. Cuvelier, Z. Alraies, M. Molina, G. P. F. Nader, N. Srivastava, P. J. Sáez, J. M. Garcia-Arcos, I. Y. Zhitnyak, A. Bhargava, M. K. Driscoll, E. S. Welf, R. Fiolka, R. J. Petrie, N. S. D. Silva, J. M. González-Granado, N. Manel, A. M. Lennon-Duménil, D. J. Müller, M. Piel, The nucleus acts as a ruler tailoring cell responses to spatial constraints. *Science*, **370** eaba2894 (2020).

10. N. F. Li, E. Gemenetzidis, F. J. Marshall, D. Davies, Y. Yu, K. Frese, F. E. M. Froeling, A. K. Woolf, R. M. Feakins, Y. Naito, C. Iacobuzio-Donahue, D. A. Tuveson, I. R. Hart, H. M. Kocher, RhoC interacts with integrin α5β1 and enhances its trafficking in migrating pancreatic carcinoma cells. *PLOS ONE* **8**, e81575 (2013).

11. A. M. Krebs, J. Mitschke, M. Lasierra Losada, O. Schmalhofer, M. Boerries, H. Busch, M. Boettcher, D. Mougiakakos, W. Reichardt, P. Bronsert, V. G. Brunton, C. Pilarsky, T. H. Winkler, S. Brabletz, M. P. Stemmler, T. Brabletz, The EMT-activator Zeb1 is a key factor for cell plasticity and promotes metastasis in pancreatic cancer. *Nat. Cell Biol.* **19**, 518–529 (2017).

12. A. D. Rhim, E. T. Mirek, N. M. Aiello, A. Maitra, J. M. Bailey, F. McAllister, M. Reichert, G. L. Beatty, A. K. Rustgi, R. H. Vonderheide, S. D. Leach, B. Z. Stanger, EMT and dissemination precede pancreatic tumor formation. *Cell* **148**, 349–361 (2012).

13. T. Armstrong, G. Packham, L. B. Murphy, A. C. Bateman, J. A. Conti, D. R. Fine, C. D. Johnson, R. C. Benyon, J. P. Iredale, Type I collagen promotes the malignant phenotype of pancreatic ductal adenocarcinoma. *Clin. Cancer Res.* **10**, 7427–7437 (2004).

14. V. Sanz-Moreno, G. Gadea, J. Ahn, H. Paterson, P. Marra, S. Pinner, E. Sahai, C. J. Marshall, Rac activation and inactivation control plasticity of tumor cell movement. *Cell* **135**, 510–523 (2008).

15. I. Rodriguez-Hernandez, G. Cantelli, F. Bruce, V. Sanz-Moreno, Rho, ROCK and actomyosin contractility in metastasis as drug targets. *F1000Research* **5**, 783 (2016).

16. O. Maiques, B. Fanshawe, E. Crosas-Molist, I. Rodriguez-Hernandez, A. Volpe, G. Cantelli, L. Boehme, J. L. Orgaz, F. K. Mardakheh, V. Sanz-Moreno, G. O. Fruhwirth, A preclinical pipeline to evaluate migrastatics as therapeutic agents in metastatic melanoma. *Br. J. Cancer* **125**, 699–713 (2021).

17. P. Timpson, E. J. McGhee, J. P. Morton, A. von Kriegsheim, J. P. Schwarz, S. A. Karim, B. Doyle, J. A. Quinn, N. O. Carragher, M. Edward, M. F. Olson, M. C. Frame, V. G. Brunton, O. J. Sansom, K. I. Anderson, Spatial regulation of RhoA activity during pancreatic cancer cell invasion driven by mutant p53. *Cancer Res.* **71**, 747–757 (2011).

18. N. Rath, J. P. Morton, L. Julian, L. Helbig, S. Kadir, E. J. McGhee, K. I. Anderson, G. Kalna, M. Mullin, A. V. Pinho, I. Rooman, M. S. Samuel, M. F. Olson, ROCK signaling promotes collagen remodeling to facilitate invasive pancreatic ductal adenocarcinoma tumor cell growth. *EMBO Mol. Med.* **9**, 198–218 (2017).

19. H. Laklai, Y. A. Miroshnikova, M. W. Pickup, E. A. Collisson, G. E. Kim, A. S. Barrett, R. C. Hill, J. N. Lakins, D. D. Schlaepfer, J. K. Mouw, V. S. LeBleu, N. Roy, S. V. Novitskiy, J. S. Johansen, V. Poli, R. Kalluri, C. A. Iacobuzio-Donahue, L. D. Wood, M. Hebrok, K. Hansen, H. L. Moses, V. M. Weaver, Genotype tunes pancreatic ductal adenocarcinoma tissue tension to induce matricellular fibrosis and tumor progression. *Nat. Med.* **22**, 497–505 (2016).

20. N. Rath, J. Munro, M. F. Cutiongco, A. Jagiełło, N. Gadegaard, L. McGarry, M. Unbekandt, E. Michalopoulou, J. J. Kamphorst, D. Sumpton, G. Mackay, C. Vennin, M. Pajic, P. Timpson, M. F. Olson, Rho kinase inhibition by AT13148 blocks pancreatic ductal adenocarcinoma invasion and tumor growth. *Cancer Res.* **78**, 3321–3336 (2018).

21. I. Rodriguez-Hernandez, O. Maiques, L. Kohlhammer, G. Cantelli, A. Perdrix-Rosell, J. Monger, B. Fanshawe, V. L. Bridgeman, S. N. Karagiannis, R. M. Penin, J. Marcolval, R. M. Marti, X. Matias-Guiu, G. O. Fruhwirth, J. L. Orgaz, I. Malanchi, V. Sanz-Moreno, WNT11-FZD7-DAAM1 signalling supports tumour initiating abilities and melanoma amoeboid invasion. *Nat. Commun.* **11**, 5315 (2020).

22. M. L. Tognoli, N. Vlahov, S. Steenbeek, A. M. Grawenda, M. Eyres, D. Cano-Rodriguez, S. Scrace, C. Kartsonaki, A. von Kriegsheim, E. Willms, M. J. Wood, M. G. Rots, J. van Rheenen, E. O'Neill, D. Pankova, RASSF1C oncogene elicits amoeboid invasion, cancer stemness, and extracellular vesicle release via a SRC/Rho axis. *EMBO J.* **40**, e107680 (2021).

23. H. Takeda, M. Okada, S. Suzuki, K. Kuramoto, H. Sakaki, H. Watarai, T. Sanomachi, S. Seino, T. Yoshioka, C. Kitanaka, Rho-associated protein kinase (ROCK) inhibitors inhibit survivin expression and sensitize pancreatic cancer stem cells to gemcitabine. *Anticancer Res* **36**, 6311–6318 (2016).

24. G. Cantelli, J. L. Orgaz, I. Rodriguez-Hernandez, P. Karagiannis, O. Maiques, X. Matias-Guiu, F. O. Nestle, R. M. Marti, S. N. Karagiannis, V. Sanz-Moreno, TGF-β-induced transcription sustains amoeboid melanoma migration and dissemination. *Curr. Biol. CB* **25**, 2899–2914 (2015).

25. J. L. Orgaz, E. Crosas-Molist, A. Sadok, A. Perdrix-Rosell, O. Maiques, I. Rodriguez-Hernandez, J. Monger, S. Mele, M. Georgouli, V. Bridgeman, P. Karagiannis, R. Lee, P. Pandya, L. Boehme, F. Wallberg, C. Tape, S. N. Karagiannis, I. Malanchi, V. Sanz-Moreno, Myosin II reactivation and cytoskeletal remodeling as a hallmark and a vulnerability in melanoma therapy resistance. *Cancer Cell* **37**, 85–103.e9 (2020).

26. M. Georgouli, C. Herraiz, E. Crosas-Molist, B. Fanshawe, O. Maiques, A. Perdrix, P. Pandya, I. Rodriguez-Hernandez, K. M. Ilieva, G. Cantelli, P. Karagiannis, S. Mele, H. Lam, D. H. Josephs, X. Matias-Guiu, R. M. Marti, F. O. Nestle, J. L. Orgaz, I. Malanchi, G. O. Fruhwirth, S. N. Karagiannis, V. Sanz-Moreno, Regional activation of Myosin II in cancer cells drives tumor progression via a secretory cross-talk with the immune microenvironment. *Cell* **176**, 757–774.e23 (2019).

27. N. Martinez-Bosch, J. Vinaixa, P. Navarro, Immune evasion in pancreatic cancer: From mechanisms to therapy. *Cancer* **10**, 6 (2018).

28. A. Ene–Obong, A. J. Clear, J. Watt, J. Wang, R. Fatah, J. C. Riches, J. F. Marshall, J. Chin–Aleong, C. Chelala, J. G. Gribben, A. G. Ramsay, H. M. Kocher, Activated pancreatic stellate cells sequester CD8+ T cells to reduce their infiltration of the juxtatumoral compartment of pancreatic ductal adenocarcinoma. *Gastroenterology* **145**, 1121–1132 (2013).

29. V. Sanz-Moreno, C. Gaggioli, M. Yeo, J. Albrengues, F. Wallberg, A. Viros, S. Hooper, R. Mitter, C. C. Féral, M. Cook, J. Larkin, R. Marais, G. Meneguzzi, E. Sahai, C. J. Marshall, ROCK and JAK1 signaling cooperate to control actomyosin contractility in tumor cells and stroma. *Cancer Cell* **20**, 229–245 (2011).

30. K. Patil, F. B. Khan, S. Akhtar, A. Ahmad, S. Uddin, The plasticity of pancreatic cancer stem cells: Implications in therapeutic resistance. *Cancer Metastasis Rev.* **40**, 691–720 (2021).

31. M. Mortoglou, F. Miralles, E. D. Arisan, A. Dart, S. Jurcevic, S. Lange, P. Uysal-Onganer, microRNA-21 regulates stemness in pancreatic ductal adenocarcinoma cells. *Int. J. Mol. Sci.* **23**, 1275 (2022).

32. Y. Wang, R. Gangeswaran, X. Zhao, P. Wang, J. Tysome, V. Bhakta, M. Yuan, C. P. Chikkanna-Gowda, G. Jiang, D. Gao, F. Cao, J. Francis, J. Yu, K. Liu, H. Yang, Y. Zhang, W. Zang, C. Chelala, Z.

Dong, N. Lemoine, CEACAM6 attenuates adenovirus infection by antagonizing viral trafficking in cancer cells. *J. Clin. Invest.* **119**, 1604–1615 (2009).

33. A. D. Weems, E. S. Welf, M. K. Driscoll, F. Y. Zhou, H. Mazloom-Farsibaf, B.-J. Chang, V. S. Murali, G. M. Gihana, B. G. Weiss, J. Chi, D. Rajendran, K. M. Dean, R. Fiolka, G. Danuser, Blebs promote cell survival by assembling oncogenic signalling hubs. *Nature* **615**, 517–525 (2023).

34. J. L. Orgaz, P. Pandya, R. Dalmeida, P. Karagiannis, B. Sanchez-Laorden, A. Viros, J. Albrengues, F. O. Nestle, A. J. Ridley, C. Gaggioli, R. Marais, S. N. Karagiannis, V. Sanz-Moreno, Diverse matrix metalloproteinase functions regulate cancer amoeboid migration. *Nat. Commun.* **5**, 4255 (2014).

35. N. S. Clayton, A. J. Ridley, Targeting Rho GTPase signaling networks in cancer. *Front. Cell Dev. Biol.* **8** 222 (2020).

36. C. Doe, R. Bentley, D. J. Behm, R. Lafferty, R. Stavenger, D. Jung, M. Bamford, T. Panchal, E. Grygielko, L. L. Wright, G. K. Smith, Z. Chen, C. Webb, S. Khandekar, T. Yi, R. Kirkpatrick, E. Dul, L. Jolivette, J. P. Marino, R. Willette, D. Lee, E. Hu, Novel Rho kinase inhibitors with anti-inflammatory and vasodilatory activities. *J. Pharmacol. Exp. Ther.* **320**, 89–98 (2007).

37. M. Dibble, S. D. Cio, P. Luo, F. Balkwill, J. E. Gautrot, Impact of pericytes on the stabilisation of microvascular networks in microfluidic systems in response to nanotoxicity, 2022.05.03.490457 (2022).

38. S. R. Hingorani, L. Wang, A. S. Multani, C. Combs, T. B. Deramaudt, R. H. Hruban, A. K. Rustgi, S. Chang, D. A. Tuveson, Trp53R172H and KrasG12D cooperate to promote chromosomal instability and widely metastatic pancreatic ductal adenocarcinoma in mice. *Cancer Cell* **7**, 469–483 (2005).

39. C. Gaggioli, S. Hooper, C. Hidalgo-Carcedo, R. Grosse, J. F. Marshall, K. Harrington, E. Sahai, Fibroblast-led collective invasion of carcinoma cells with differing roles for RhoGTPases in leading and following cells. *Nat. Cell Biol.* **9**, 1392–1400 (2007).

40. B. Erdogan, M. Ao, L. M. White, A. L. Means, B. M. Brewer, L. Yang, M. K. Washington, C. Shi, O. E. Franco, A. M. Weaver, S. W. Hayward, D. Li, D. J. Webb, Cancer-associated fibroblasts promote directional cancer cell migration by aligning fibronectin. *J. Cell Biol.* **216**, 3799–3816 (2017).

41. S. R. Nielsen, V. Quaranta, A. Linford, P. Emeagi, C. Rainer, A. Santos, L. Ireland, T. Sakai, K. Sakai, Y.-S. Kim, D. Engle, F. Campbell, D. Palmer, J. H. Ko, D. A. Tuveson, E. Hirsch, A. Mielgo, M. C. Schmid, Macrophage-secreted granulin supports pancreatic cancer metastasis by inducing liver fibrosis. *Nat. Cell Biol.* **18**, 549–560 (2016).

42. D. Vijayan, A. Young, M. W. L. Teng, M. J. Smyth, Targeting immunosuppressive adenosine in cancer. *Nat. Rev. Cancer* **17**, 709–724 (2017).

43. M. Lupia, F. Angiolini, G. Bertalot, S. Freddi, K. F. Sachsenmeier, E. Chisci, B. Kutryb-Zajac, S. Confalonieri, R. T. Smolenski, R. Giovannoni, N. Colombo, F. Bianchi, U. Cavallaro, CD73 regulates stemness and epithelial-mesenchymal transition in ovarian cancer-initiating cells. *Stem Cell Rep.* **10**, 1412–1425 (2018).

44. N. Petruk, S. Tuominen, M. Åkerfelt, J. Mattsson, J. Sandholm, M. Nees, G. G. Yegutkin, A. Jukkola, J. Tuomela, K. S. Selander, CD73 facilitates EMT progression and promotes lung metastases in triple-negative breast cancer. *Sci. Rep.* **11**, 6035 (2021).

45. Z. Xu, C. Gu, X. Yao, W. Guo, H. Wang, T. Lin, F. Li, D. Chen, J. Wu, G. Ye, L. Zhao, Y. Hu, J. Yu, J. Shi, G. Li, H. Liu, CD73 promotes tumor metastasis by modulating RICS/RhoA signaling and EMT in gastric cancer. *Cell Death Dis.* **11**, 1–15 (2020).

46. X.-L. Ma, B. Hu, W.-G. Tang, S.-H. Xie, N. Ren, L. Guo, R.-Q. CD73 sustained cancer-stem-cell traits by promoting SOX9 expression and stability in hepatocellular *carcinoma.J. Hematol. Oncol.* **13**, 11 (2020).

47. L. Zhou, S. Jia, Y. Chen, W. Wang, Z. Wu, W. Yu, M. Zhang, G. Ding, L. Cao, The distinct role of CD73 in the progression of pancreatic cancer. *J. Mol. Med. Berl. Ger.* **97**, 803–815 (2019).

48. C. J. Vlahos, W. F. Matter, K. Y. Hui, R. F. Brown, A specific inhibitor of phosphatidylinositol 3-kinase, 2-(4-morpholinyl)-8-phenyl-4H-1-benzopyran-4-one (LY294002). *J. Biol. Chem.* **269**, 5241–5248 (1994).

49. W. Lin, P. Noel, E. H. Borazanci, J. Lee, A. Amini, I. W. Han, J. S. Heo, G. S. Jameson, C. Fraser, M. Steinbach, Y. Woo, Y. Fong, D. Cridebring, D. D. Von Hoff, J. O. Park, H. Han, Single-cell

transcriptome analysis of tumor and stromal compartments of pancreatic ductal adenocarcinoma primary tumors and metastatic lesions. *Genome Med.* **12**, 80 (2020).

50. A. M. Newman, C. L. Liu, M. R. Green, A. J. Gentles, W. Feng, Y. Xu, C. D. Hoang, M. Diehn, A. A. Alizadeh, Robust enumeration of cell subsets from tissue expression profiles. *Nat. Methods* **12**, 453–457 (2015).

51. R. A. Moffitt, R. Marayati, E. L. Flate, K. E. Volmar, S. G. H. Loeza, K. A. Hoadley, N. U. Rashid, L. A. Williams, S. C. Eaton, A. H. Chung, J. K. Smyla, J. M. Anderson, H. J. Kim, D. J. Bentrem, M. S. Talamonti, C. A. Iacobuzio-Donahue, M. A. Hollingsworth, J. J. Yeh, Virtual microdissection identifies distinct tumor- and stroma-specific subtypes of pancreatic ductal adenocarcinoma. *Nat. Genet.* **47**, 1168–1178 (2015).

52. N. Iwai, T. Okuda, J. Sakagami, T. Harada, T. Ohara, M. Taniguchi, H. Sakai, K. Oka, T. Hara, T. Tsuji, T. Komaki, K. Kagawa, H. Yasuda, Y. Naito, Y. Itoh, Neutrophil to lymphocyte ratio predicts prognosis in unresectable pancreatic cancer. *Sci. Rep.* **10**, 18758 (2020).

53. S. Yachida, S. Jones, I. Bozic, T. Antal, R. Leary, B. Fu, M. Kamiyama, R. H. Hruban, J. R. Eshleman, M. A. Nowak, V. E. Velculescu, K. W. Kinzler, B. Vogelstein, C. A. Iacobuzio-Donahue, Distant metastasis occurs late during the genetic evolution of pancreatic cancer. *Nature* **467**, 1114–1117 (2010).

54. A. Reversat, F. Gaertner, J. Merrin, J. Stopp, S. Tasciyan, J. Aguilera, I. de Vries, R. Hauschild, M. Hons, M. Piel, A. Callan-Jones, R. Voituriez, M. Sixt, Cellular locomotion using environmental topography. *Nature* , **582** (2020), 582, 585.

55. J. Ahn, V. Sanz-Moreno, C. J. Marshall, The metastasis gene NEDD9 product acts through integrin β3 and Src to promote mesenchymal motility and inhibit amoeboid motility. *J. Cell Sci.* **125**, 1814–1826 (2012).

56. S. Giampieri, C. Manning, S. Hooper, L. Jones, C. S. Hill, E. Sahai, Localized and reversible TGFbeta signalling switches breast cancer cells from cohesive to single cell motility. *Nat. Cell Biol.* **11**, 1287–1296 (2009).

57. D. L. Moose, B. L. Krog, T.-H. Kim, L. Zhao, S. Williams-Perez, G. Burke, L. Rhodes, M. Vanneste, P. Breheny, M. Milhem, C. S. Stipp, A. C. Rowat, M. D. Henry, Cancer cells resist mechanical destruction in circulation via rhoa/actomyosin-dependent mechano-adaptation. *Cell Rep.* **30**, 3864–3874.e6 (2020).

58. D. Schizas, N. Charalampakis, C. Kole, P. Economopoulou, E. Koustas, E. Gkotsis, D. Ziogas, A. Psyrri, M. V. Karamouzis, Immunotherapy for pancreatic cancer: A 2020 update. *Cancer Treat. Rev.* **86**, 102016 (2020).

59. R. Turiello, M. Capone, E. Morretta, M. C. Monti, G. Madonna, R. Azzaro, P. D. Gaudio, E. Simeone, A. Sorrentino, P. A. Ascierto, S. Morello, Exosomal CD73 from serum of patients with melanoma suppresses lymphocyte functions and is associated with therapy resistance to anti-PD-1 agents. *J. Immunother. Cancer* **10**, e004043 (2022).

60. A. Young, S. F. Ngiow, D. S. Barkauskas, E. Sult, C. Hay, S. J. Blake, Q. Huang, J. Liu, K. Takeda, M. W. L. Teng, K. Sachsenmeier, M. J. Smyth, Co-inhibition of CD73 and A2AR adenosine signaling improves anti-tumor immune responses. *Cancer Cell* **30**, 391–403 (2016).

61. X. Yu, W. Liu, Z. Wang, H. Wang, J. Liu, C. Huang, T. Zhao, X. Wang, S. Gao, Y. Ma, L. Wu, X. Li, S. Yang, J. Hao, CD73 induces gemcitabine resistance in pancreatic ductal adenocarcinoma: A promising target with non-canonical mechanisms. *Cancer Lett.* **519**, 289–303 (2021).

62. X.-L. Ma, M.-N. Shen, B. Hu, B.-L. Wang, W.-J. Yang, L.-H. Lv, H. Wang, Y. Zhou, A.-L. Jin, Y.-F. Sun, C.-Y. Zhang, S.-J. Qiu, B.-S. Pan, J. Zhou, J. Fan, X.-R. Yang, W. Guo, CD73 promotes hepatocellular carcinoma progression and metastasis via activating PI3K/AKT signaling by inducing Rap1-mediated membrane localization of P110β and predicts poor *prognosis.J. Hematol. Oncol.* **12**, 37 (2019).

63. B. K. Tripathi, T. Grant, X. Qian, M. Zhou, P. Mertins, D. Wang, A. G. Papageorge, S. G. Tarasov, K. W. Hunter, S. A. Carr, D. R. Lowy, Receptor tyrosine kinase activation of RhoA is mediated by AKT phosphorylation of DLC1. *J. Cell Biol.* **216**, 4255–4270 (2017).

64. S. Deaglio, K. M. Dwyer, W. Gao, D. Friedman, A. Usheva, A. Erat, J.-F. Chen, K. Enjyoji, J. Linden, M. Oukka, V. K. Kuchroo, T. B. Strom, S. C. Robson, Adenosine generation catalyzed by CD39 and CD73 expressed on regulatory T cells mediates immune suppression. *J. Exp. Med.* **204**, 1257–1265 (2007).

65. F. Calvo, N. Ege, A. Grande-Garcia, S. Hooper, R. P. Jenkins, S. I. Chaudhry, K. Harrington, P. Williamson, E. Moeendarbary, G. Charras, E. Sahai, Mechanotransduction and YAP-dependent matrix remodelling is required for the generation and maintenance of cancer-associated fibroblasts. *Nat. Cell Biol.* **15**, 637–646 (2013).

66. Y. Attieh, A. G. Clark, C. Grass, S. Richon, M. Pocard, P. Mariani, N. Elkhatib, T. Betz, B. Gurchenkov, D. M. Vignjevic, Cancer-associated fibroblasts lead tumor invasion through integrin-β3–dependent fibronectin assembly. *J. Cell Biol.* **216**, 3509–3520 (2017).

67. C. Vennin, V. T. Chin, S. C. Warren, M. C. Lucas, D. Herrmann, A. Magenau, P. Melenec, S. N. Walters, G. del Monte-Nieto, J. R. W. Conway, M. Nobis, A. H. Allam, R. A. McCloy, N. Currey, M. Pinese, A. Boulghourjian, A. Zaratzian, A. A. S. Adam, C. Heu, A. M. Nagrial, A. Chou, A. Steinmann, A. Drury, D. Froio, M. Giry-Laterriere, N. L. E. Harris, T. Phan, R. Jain, W. Weninger, E. J. McGhee, R. Whan, A. L. Johns, J. S. Samra, L. Chantrill, A. J. Gill, M. Kohonen-Corish, R. P. Harvey, A. V. Biankin, A. P. C. G. Initiative (APGI), T. R. J. Evans, K. I. Anderson, S. T. Grey, C. J. Ormandy, D. Gallego-Ortega, Y. Wang, M. S. Samuel, O. J. Sansom, A. Burgess, T. R. Cox, J. P. Morton, M. Pajic, P. Timpson, Transient tissue priming via ROCK inhibition uncouples pancreatic cancer progression, sensitivity to chemotherapy, and metastasis. *Sci. Transl. Med.* **9** 1 (2017).

68. A. Glentis, P. Oertle, P. Mariani, A. Chikina, F. El Marjou, Y. Attieh, F. Zaccarini, M. Lae, D. Loew, F. Dingli, P. Sirven, M. Schoumacher, B. G. Gurchenkov, M. Plodinec, D. M. Vignjevic, Cancer-associated fibroblasts induce metalloprotease-independent cancer cell invasion of the basement membrane. *Nat. Commun.* **8**, 924 (2017).

69. E. Elyada, M. Bolisetty, P. Laise, W. F. Flynn, E. T. Courtois, R. A. Burkhart, J. A. Teinor, P. Belleau, G. Biffi, M. S. Lucito, S. Sivajothi, T. D. Armstrong, D. D. Engle, K. H. Yu, Y. Hao, C. L. Wolfgang, Y. Park, J. Preall, E. M. Jaffee, A. Califano, P. Robson, D. A. Tuveson, Cross-species single-cell

analysis of pancreatic ductal adenocarcinoma reveals antigen-presenting cancer-associated fibroblasts. *Cancer Discov.*, **9** 1102–1123 (2019).

70. J. Barcelo, R. Samain, V. Sanz-Moreno, Preclinical to clinical utility of ROCK inhibitors in cancer. *Trends Cancer* **9**, 250–263 (2023).

71. J. B. Wyckoff, S. E. Pinner, S. Gschmeissner, J. S. Condeelis, E. Sahai, ROCK- and myosin-dependent matrix deformation enables protease-independent tumor-cell invasion in vivo. *Curr. Biol.* **16**, 1515–1523 (2006).

72. O. Maiques, V. Sanz-Moreno, Multiplex chromogenic immunohistochemistry to stain and analyze paraffin tissue sections from the mouse or human. *STAR Protoc.* **3**, 101879 (2022).

73. G. Zhang, A. Schetter, P. He, N. Funamizu, J. Gaedcke, B. M. Ghadimi, T. Ried, R. Hassan, H. G. Yfantis, D. H. Lee, C. Lacy, A. Maitra, N. Hanna, H. R. Alexander, S. P. Hussain, DPEP1 inhibits tumor cell invasiveness, enhances chemosensitivity and predicts clinical outcome in pancreatic ductal adenocarcinoma. *PLOS ONE* **7**, e31507 (2012).

74. G. Bindea, B. Mlecnik, H. Hackl, P. Charoentong, M. Tosolini, A. Kirilovsky, W.-H. Fridman, F. Pagès, Z. Trajanoski, J. Galon, ClueGO: A Cytoscape plug-in to decipher functionally grouped gene ontology and pathway annotation networks. *Bioinformatics* **25**, 1091–1093 (2009).

75. U. Raudvere, L. Kolberg, I. Kuzmin, T. Arak, P. Adler, H. Peterson, J. Vilo, g:Profiler: A web server for functional enrichment analysis and conversions of gene lists (2019 update). *Nucleic Acids Res.* **47**, W191–W198 (2019).

76. G. Tang, M. Cho, X. Wang, OncoDB: An interactive online database for analysis of gene expression and viral infection in cancer. *Nucleic Acids Res.* **50**, D1334–D1339 (2022).

77. D. Sun, J. Wang, Y. Han, X. Dong, J. Ge, R. Zheng, X. Shi, B. Wang, Z. Li, P. Ren, L. Sun, Y. Yan, P. Zhang, F. Zhang, T. Li, C. Wang, TISCH: A comprehensive web resource enabling interactive single-cell transcriptome visualization of tumor microenvironment. *Nucleic Acids Res.* **49**, D1420–D1430 (2021).
